# Implementing biofeedback treatment in a psychosomatic-psychotherapeutic inpatient unit: a mixed methods evaluation of acceptance, satisfaction, and feasibility

**DOI:** 10.3389/fpsyt.2023.1140880

**Published:** 2023-05-24

**Authors:** Kira Schmidt, Drazena Barac-Dammeyer, Axel Kowalski, Per Teigelack, Corinna Pfeiffer, Anita Robitzsch, Nora Dörrie, Eva-Maria Skoda, Alexander Bäuerle, Madeleine Fink, Martin Teufel

**Affiliations:** ^1^Department for Psychosomatic Medicine and Psychotherapy, LVR-University Hospital Essen, University of Duisburg-Essen, Essen, Germany; ^2^Center for Translational Neuro-and Behavioral Sciences (C-TNBS), University of Duisburg-Essen, Essen, Germany; ^3^NeuroFit GmbH, Krefeld, Germany; ^4^Department of Psychology, IB University of Applied Health and Social Sciences, Berlin, Germany

**Keywords:** biofeedback, neurofeedback, psychosomatic, implementation, mixed methods, inpatient

## Abstract

**Introduction:**

Feedback-based therapies such as biofeedback have a benefit in patients with mental health disorders. While biofeedback is heavily researched in outpatient settings, it has been rarely investigated in psychosomatic inpatient settings. The implementation of an additional treatment option in inpatient settings holds special requirements. The aim of this pilot study is the evaluation of additional biofeedback treatment in an inpatient psychosomatic-psychotherapeutic unit to derive clinical implications and recommendations for the future implementation of biofeedback offers.

**Methods:**

The evaluation of the implementation process was investigated using a convergent parallel mixed methods approach (following MMARS guidelines). Quantitative questionnaires measured patients’ acceptance and satisfaction with biofeedback treatment after receiving 10 sessions in addition to treatment as usual. After 6 months during implementation, qualitative interviews were conducted with biofeedback practitioners, i.e., staff nurses, examining acceptance and feasibility. Data analysis was conducted using either descriptive statistics or Mayring’s qualitative content analysis.

**Results:**

In total, 40 patients and 10 biofeedback practitioners were included. Quantitative questionnaires revealed high satisfaction and acceptance in patients regarding biofeedback treatment. Qualitative interviews showed high acceptance in biofeedback practitioners but revealed several challenges that were encountered during the implementation process, e.g., increased workload due to additional tasks, organizational and structural difficulties. However, biofeedback practitioners were enabled to expand their own competencies and take over a therapeutic part of the inpatient treatment.

**Discussion:**

Even though patient satisfaction and staff motivation are high, the implementation of biofeedback in an inpatient unit requires special actions to be taken. Not only should personnel resources be planned and available in advance of implementation but also be the workflow for biofeedback practitioners as easy and quality of biofeedback treatment as high as possible. Consequently, the implementation of a manualized biofeedback treatment should be considered. Nevertheless, more research needs to be done about suitable biofeedback protocols for this patient clientele.

## 1. Introduction

Feedback is an essential component in psychotherapeutic interventions: it facilitates learning, increases motivation, and modifies thoughts or behavior (1). Biofeedback (BFB) as psychophysiological therapy is taught through cognitive changes (2), such as improving self-efficacy (3) or learning coping strategies. Typically, BFB is provided on parameters like muscle tone, respiratory rate, heart rate, skin conductance, skin surface temperature, or brain activity (i.e., neurofeedback, NFB) (4). The effectiveness of BFB has been investigated in several somatic and mental health disorders. The most common use of feedback-based therapies is in fields such as epilepsy (5), migraine (6), strokes (7), attention deficit / hyperactivity disorder (8), autism spectrum disorder (9), major depression and anxiety disorders (10), as well as addiction (11) and psychotic disorders (12). However, BFB has been rarely investigated in the specialty of psychosomatic medicine, the core idea of which is that mind and body both contribute an essential part to human function and which represents an independent specialty in Germany (13, 14). In the context of psychosomatic illnesses (i.e., somatoform/functional disorders, somatopsychic disorders, eating disorders, posttraumatic stress disorders, depressive disorders), several studies examined the use of BFB, e.g., to affect pain perception. In a patient case study, duration of the headaches and decreased intensity were related to increased alpha activity (8–12 Hz) (15). Glombiewski et al. could show in their meta-analysis on BFB including seven studies (321 patients) that BFB training significantly reduced pain intensity compared to controls with a large effect (2). NFB has shown promise in alleviating overall symptoms of posttraumatic stress disorder (PTSD) (16). Especially patients with PTSD who did not respond to previous treatments were able to benefit from NFB (16). Moreover, BFB had a positive impact on several eating disorders (e.g., food craving, rumination) (17). Not only were feedback-based techniques related to significant modifications in sympathetic responses to food stimuli but also to brain activity in different areas of the reward system (17).

Overall, current literature suggests a benefit of BFB and/or NFB in patients with various psychosomatic illnesses. However, while BFB has already been heavily researched in the outpatient setting (18–21), the usage of BFB and/or NFB in an inpatient setting for mental health disorders has been rarely investigated. To our knowledge, only one study examined the usage of BFB in inpatients with eating disorders (22). Another study investigated the use of BFB in combat veterans suffering from PTSD (23).

Noteworthy, the demands on a therapy offer in the inpatient setting differ from those in the outpatient setting. While the outpatient setting is characterized by weekly therapy sessions, the inpatient setting consists of several different daily therapy offers provided over several weeks. This leads to tightly scheduled appointments and therapies and units might be understaffed. This leads to higher organizational demands, which can affect the implementation and feasibility of an additional treatment offer such as BFB. Up to now, there are no studies examining the implementation process of BFB treatment in psychosomatic-psychotherapeutic inpatient settings, the associated challenges, and the acceptance of patients and BFB practitioners.

Within the scope of this pilot study, patients in our psychosomatic-psychotherapeutic unit received regular BFB treatment sessions in addition to the treatment as usual (TAU). We will then examine quantitatively the acceptance and satisfaction of patients with BFB treatment. Moreover, we will use qualitative interviews to investigate the acceptance of BFB practitioners and feasibility in conducting BFB treatment. We will then combine these methods to evaluate the ability to implement this new treatment offer into our psychosomatic-psychotherapeutic inpatient settings in a mixed methods investigation.

The aim of this pilot study is the evaluation of additional BFB treatment in an inpatient psychosomatic-psychotherapeutic unit by investigating acceptance, satisfaction, and feasibility of BFB treatment. On this basis, we will derive clinical implications and develop recommendations for action for the future implementation of BFB offers in psychosomatic-psychotherapeutic inpatient settings.

## 2. Method

To assess acceptance, satisfaction, and feasibility of a BFB treatment in addition to the TAU, we conducted a convergent parallel mixed methods approach, which involves collecting qualitative and quantitative data simultaneously, and then combining and comparing these multiple data sources (24), by using quantitative questionnaires for patients as well as qualitative semi-structured interviews for BFB practitioners. Both quantitative and qualitative data were collected parallel during the implementation process and analyzed separately. Integration of the results will lead to a more comprehensive evaluation of the implementation process. In conducting and reporting this pilot study we followed the *Mixed Methods Article Reporting Standards (MMARS)* by the American Psychological Association (25). Evaluation was granted exemption from ethical review by the Ethics Committee of the Medical Faculty of the University of Duisburg-Essen (No. 19-8893-BO). The study was conducted in accordance with the Declaration of Helsinki.

### 2.1. Study design and procedure

Implementation of the BFB treatment started in November 2021 as an additional routine treatment program in the inpatient unit of the LVR-University Hospital, Clinic for Psychosomatic Medicine and Psychotherapy (for further information regarding inpatient therapy see below in section 2.2 Setting or above in the introduction). Prior to this, the equipment was set up and instructions and manuals were created to assist BFB practitioners, i.e., staff nurses, in delivering the treatment. Due to the pilot character of this study, the BFB was an ordered new activity, for which the BFB practitioners received valences. The implementation started with a training of the BFB practitioners by the project leader (PL). Technical as well as theoretical basics were explained, and the conduction of sessions was exemplified and practiced. The BFB practitioners and the PL were supervised by a certified BFB instructor (author AK). Recruitment of patients took place during the first week of their admission to the inpatient unit *via* an information sheet. If patients expressed interest, they were referred to the PL, who then conducted the educational interview and obtained written informed consent. Inclusion criteria were a psychosomatic diagnosis (i.e., somatoform/functional disorders, somatopsychic disorders, eating disorders, posttraumatic stress disorders, depressive disorders), written informed consent, and age between 18 and 70 years. Exclusion criteria were neurological or central nervous disorders or insufficient language skills. BFB treatment appointments were made individually with patients by the nursing staff or PL. BFB sessions were conducted between November 25, 2021, and August 03, 2022. During the BFB treatment, different BFB practitioners conducted BFB sessions with each patient. Patients received 10 sessions of BFB treatment twice a week over a period of 5 weeks. After the last session, patients were asked to answer self-report questionnaires and they were given the opportunity to attend a follow-up meeting with the PL to clarify open questions. On March 1, 2022, a student assistant helped in assistance with the implementation and execution of the sessions. After 6 months following the start of implementation, the PL conducted qualitative semi-structured interviews with all BFB practitioners after they gave written informed consent.

### 2.2. Setting

The study site was an inpatient unit providing care for adults aged 18–70 years, within a psychosomatic-psychotherapeutic clinic in a university hospital. Patients either remained as full inpatients or stayed overnight in the clinic or spent only the therapy day in the clinic as part-time inpatients. The length of stay inpatient ranges from 6 weeks to 3 months. The psychosomatic diagnoses of patients vary and include eating disorders and obesity, somatoform disorders, PTSD, psycho-cardiologic, and affective diagnoses. Patients receive a comprehensive therapy program (i.e., TAU) ranging from therapeutically guided interactional group therapy and individual sessions to psychoeducation, art therapy, expressive painting, skills groups, sports and movement therapy, needs-oriented social work discussions, regular nursing individual sessions, medical care by ward physicians and regular visits by senior physicians (see A1 example schedule in the supplements). The BFB was offered in addition to the TAU.

### 2.3. BFB intervention

The BFB treatment was conducted using the NeXus-10B Set device and the corresponding software BioTrace (Mind Media, 2022) to collect psychophysiological information for BFB with a total of 10 channels. Trained parameters varied depending on the psychosomatic diagnosis and individually reported symptoms based on previous literature. Patients with somatoform disorders received electromyographic BFB (2) with positioning the electrodes in the neck-shoulder area. Patients with affective disorders, eating disorders or obesity, or PTSD received NFB treatment (16, 17, 26–28) with positioning the electrode on coordinate Cz and conducting alpha-frequency training to cause a relaxed brain state as well as theta-and beta-reduction to reduce arousal. Patients with psycho-cardiologic disorders received either electromyographic BFB or heartrate-variability training (29). The treatment took place in a multipurpose room, which was not used for other purposes during BFB sessions. Patients sat on a relaxation chair in distance of 1.5 m to the screen. As stimuli, patients saw a puzzle or a relaxing video, which continued depending on the degree of match with the target condition, i.e., reduction of muscle tone, increase of alpha-and reduction of theta- and beta-frequency, or increase of heartrate variability. Trained parameters were displayed as colored bar charts on the left side of the screen. The performing staff was present during the training but did not manipulate the feedback process or give verbal feedback. Patients were given a printout of every session to take it home. The BFB intervention comprised 10 training sessions, each lasting 40–45 min, taking place in the inpatient unit.

### 2.4. Measurement instruments

Data were collected using (a) anonymous quantitative self-report questionnaires answered by patients and (b) qualitative semi-structured interviews answered by the performing staff.

#### 2.4.1. Quantitative data collection of patients

The quantitative questionnaire consisted of sociodemographic items, which were collected during patient admission (i.e., questionnaires in the preparation phase), as well as validated instruments answered by the patients after their last BFB session. The patients’ satisfaction with the received BFB treatment was measured according to the Patient Satisfaction Questionnaire (ZUF-8) using 8 items on a 4-point Likert Scale, which results in a theoretical scale range of 8 to 32 ((30); see supplements A2, A3). It has a high internal consistency with Cronbach’s α = 0.90 (31). Kriz et al. defined a cut-off value of 23.5 in a psychosomatic cohort indicating high satisfaction (31). An adaptation of the System Usability Scale (SUS) was used to measure the usability of the BFB treatment with 10 items on a 5-point Likert Scale (32) (see supplements A4, A5). Reliability analysis indicated an acceptable internal consistency with Cronbach’s α = 0.70.

Based on Bangor, Kortum, and Miller, the SUS score can be translated into acceptance ranges with scores of about 73% representing good acceptance, 85% representing excellent acceptance, and 100% representing the best imaginable acceptance, respectively (33). However, acceptance can be assumed of scores >63 (33). Furthermore, acceptance and feasibility were measured with a self-generated questionnaire containing 9 items (see supplements A6, A7). Items no. 1, 2, 4, and 5 were rated on a 6-point Likert scale (very displeasing, displeasing, partly displeasing, partly pleasing, pleasing, very pleasing). Items no. 3, 6, 7, 8, and 9 were rated on a 5-point Likert scale (not applies at all, rather not applies, partly true, rather true, totally true).

#### 2.4.2. Qualitative data collection of BFB practitioners

The qualitative semi-structured interviews consisted of 12 interview questions to obtain a detailed opinion and possible suggestions for improvement of the BFB treatment offer from the BFB practitioners (see supplements A8, A9). The interview questions were derived based on the objectives of the study, which were to examine BFB practitioners’ acceptance and feasibility of conducting BFB treatment, and to identify problems and obstacles as well as develop opportunities for improvement. The interviews took between 6 and 17 min and were conducted by the PL. All interviews were audio-recorded and transcribed verbatim.

### 2.5. Data analysis

#### 2.5.1. Quantitative data analysis

Statistical analyses of quantitative data were performed using the Statistical Program for Social Sciences SPSS version 26 (IBM, New York). Figures were created using the R packages likert and ggplot. After identifying outliers (± 1 SD) *via* boxplots and exclusion from the dataset, the analyses were conducted. Simple descriptive statistics and internal consistency were computed for all quantitative questionnaire data. Acceptance and feasibility were described using frequencies.

#### 2.5.2. Qualitative data analysis

All qualitative interviews were transcribed verbatim and then served as the foundation for consecutive data analysis. The software MAXQDA 2022 (Verbi Software, 2019) was used for qualitative data analysis. All interviews were analyzed using Mayring’s method of structured content analysis (34). First, an initial deductive category system was derived from the semi-structured interview guideline. Then, in order to develop the further category system, two analyzing researchers (author 1 and author 2) coded two interviews. These researchers differed in age to enable different perspectives of the content analysis. After discussing the developed category system, it was used as a basis for coding all interviews. During the analysis, the researchers independently added, removed, or changed categories based on the text material. Until saturation of the category system was reached, relevant but still missing categories were added inductively. All interview quotations were translated from German into English language for publication purpose (for original see supplements A10).

## 3. Results

### 3.1. Quantitative patient questionnaires

#### 3.1.1. Sample characteristics

A total of 40 psychosomatic patients were included to start BFB treatment (23 female, 17 male) with a mean age of 46.28 years (SD = 13.95, median = 49.0, range: 21–69). Table 1 shows the demographic information. Due to sudden and earlier discharge of the clinic, seven patients were not able to fill in the questionnaires after BFB treatment. Moreover, four patients discontinued due to a lack of motivation and understanding the therapy concept. However, based on the pilot character of this study, demographic data of all included subjects will be reported. Subjects had various psychosomatic disorders (see Table 1). Each patient completed at least one BFB session, with an average of 8.63 sessions attended (range 1–10). 65% of the patients completed all 10 BFB sessions. See A11 in supplementary material for the type of feedback each patient received.

**Table 1:** Demographic data of patients.

	*n*	Percentage (%)
Gender		
Female	23	57.5
Male	17	42.5
Marital status		
Single	12	30
Partnership	3	7.5
Married	19	47.5
Divorced	3	7.5
Living situation		
Alone	9	22.5
With partner	16	40
Alone with child(ren)	1	2.5
With partner and child(ren)	9	22.5
With parents	1	2.5
Other	1	2.5
Education		
High school diploma	24	60
Secondary school degree („*Realschule*“)	10	25
Secondary school degree („*Hauptschule*“)	2	5
Special-needs school	1	2.5
Missing	3	7.5
Employment status		
Employed	29	72.5
Unemployed	5	12.5
Retired	3	7.5
Missing	3	7.5
Sick leave		
Yes	17	42.5
No	19	47.5
Missing	4	10
Psychosomatic disorder		
Somatoform disorder (F45.x)	11	27.5
Depression (F32.x/F33.x)	12	30
Psychocardiologic disorder (F45.30)	11	27.5
Posttraumatic stress disorder (F43.1)	3	7.5
Eating disorder (F50.x)	2	5
Obesity (E66.x)	1	2.5
*N* = 40		

#### 3.1.2. Patient satisfaction and acceptance

Table 2 shows the descriptive statistics of the quantitative data. Patients’ satisfaction with BFB ranged from 15 to 32, with a mean of 24.29 (SD = 3.87), which exceeds the cut-off value of 23.5 (31). In our sample, 18 patients exceeded the cut-off value of 23.5, six patients showed values below the cutoff. The internal consistency of the adapted ZUF-8 in our sample was excellent, with Cronbach’s α =0.903. The perceived system usability ranged from 55 to 97.5, with a mean of 72.32 (SD = 11.84).

**Table 2:** Descriptive statistics of the quantitative self-report questionnaires ZUF-8, SUS, and acceptance and feasibility answered by patients.

Outcome		*N*	*M*	*SD (SE)*
ZUF-8		24	24.29	3.87 (0.791)
SUS		29	72.32	11.84 (2.2)
Acceptance & Feasibility	1. “I found the processing of the questionnaires in the preparation phase to be...”	29	3.41	0.825 (0.153)
	2. “I found the intervention to be...”	33	3.52	0.906 (0.158)
	3. “The intervention was feasible for me without any problems.”	33	3.18	0.769 (0.134)
	4. “I found the challenges of the session to be...”	33	3.76	0.830 (0.145)
	5. “I found the basic conditions during the intervention to be...”	34	3.53	0.929 (0.159)
	6. “I found the intervention to be helpful in distracting from thoughts.”	32	2.69	0.931 (0.165)
	7. “I found it helpful to be able to take the documentation of the session with me on paper.”	30	1.83	1.289 (0.235)
	8. “I would like to continue a biofeedback offering at home.”	33	2.03	1.237 (0.215)
	9. “I would recommend this study.”	32	2.75	1.016 (0.180)

Measurement of patients’ experiences regarding BFB training. ZUF-8, Patient Satisfaction Questionnaire, SUS, System Usability Scale. All analyses were conducted outlier corrected.

**Figure 1:**
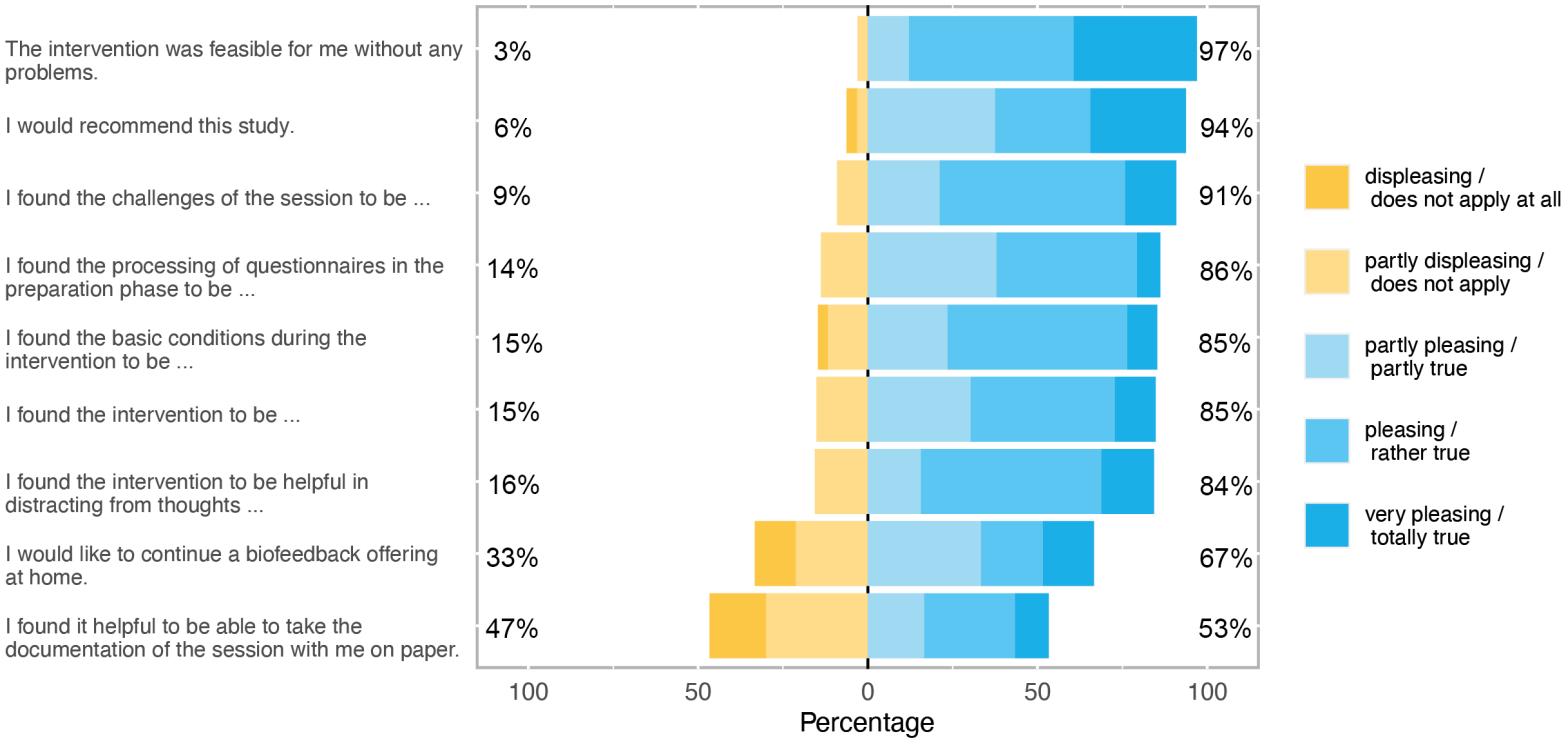
Evaluation of the self-generated questionnaire regarding patients’ acceptance and feasibility of biofeedback treatment. Deviations in the direction of approval and rejection are shown on the right and left in percental frequencies. Percentages have been rounded to whole numbers. Items appear in order of approval.

Figure 1 shows the evaluation of the self-generated questionnaire regarding acceptance and feasibility. 86.2% of the patients found the preparation of the questionnaires in the beginning as at least partly pleasant. Most of the patients (84.8%) perceived the intervention as at least partly pleasant. Only 15.2% of the patients perceived the intervention as unpleasant. 97% of the patients also indicated that the intervention had been at least partially feasible without problems. Only three patients (9.1%) indicated that the challenges of the sessions were partly unpleasant. Almost all patients (97.1%) perceived the basic conditions during the sessions as at least partly pleasant. Most of the patients (84.3%) found the intervention at least partially helpful in distracting from thoughts. 53.4% found it at least partially helpful to take the results of the session home. Most of the patients (66.7%) would like to continue BFB training at home. 93.7% of the patients at least partially recommended BFB.

### 3.2. Qualitative interviews with BFB practitioners

In total, 10 BFB practitioners (i.e., staff nurses) were involved in the implementation process of BFB by either conduction of sessions, planning BFB appointments, or managing ward-related processes in order to facilitate conduction of BFB. All of them were interviewed, nobody declined the interview. The student assistant was not interviewed to avoid conflict of interest due to involvement in project organization. Due to data privacy, we did not gather sociodemographic information of the BFB practitioners of the clinic. The duration of the interviews took between 6 and 17 min (mean 8 min 59.8 s, SD 3 min 36 s).

#### 3.2.1. Positive feedback to BFB implementation

During the interviews, different positive aspects of the implementation were mentioned. For example, the relationship with patients could be positively influenced. Moreover, an effect of the therapy on the patients’ symptoms were observed. Furthermore, the technical equipment was rated as very good. Four of 10 BFB practitioners felt supported during implementation. Supporting factors were instructions and manuals, supervisors, and colleagues as well as student assistants.

*“On the contrary, I would even say that (there’s) curiosity; this method is not known by everyone, and that this method enabled another contact with the patients.”* (Interview 8: 11).

Moreover, most BFB practitioners (8 of 10) were able to expand their area of expertise since a new therapy method was learned.

*“Yes, definitely. It’s been a new therapy method for me. I think it’s great, it’s evidence-based.”* (Interview 8: 15).

#### 3.2.2. Suitability of the BFB treatment

The majority of BFB practitioners (9 out of 10) felt that BFB treatment was suitable for the services offered in our wards. They stated it was not a replacement for traditional services, but a supplement.

*“It’s a good complement to the therapies that are offered here.”* (Interview 8: 3).

One BFB practitioner considered BFB treatment not suitable for the patient group of this setting.

#### 3.2.3. Obstacles and barriers

##### 3.2.3.1. Technical difficulties

Seven BFB practitioners named technical problems due to high complexity. Occasionally software problems occurred in the presentation of the program as well as error messages. Furthermore, the setup of the BFB device required specific knowledge. In addition, data saving and printing of the session were prone to error.

*“Yes, sometimes in the technical area, where the presentation of the program is not good in that something could not be seen on the monitor, where we then somehow also first clicked around a bit, until then you came to what you actually know.”* (Interview 4: 5).

##### 3.2.3.2. Limits of own competencies

Three BFB practitioners reached the limits of their own competencies when they did not perform BFB treatment for an extended period of time due to illness or vacation. Changes in the process were thus difficult to integrate.

*“Well, I was now and then away for longer periods of time during the time of introduction, so I was not engaged with it. I noticed that it (is) difficult to get back into it when I have not done it for a while.”* (Interview 3: 7).

Limits to one’s own competencies were also evident regarding computer skills (3 of 10 BFB practitioners).

##### 3.2.3.3. Structural difficulties

Structural problems arose in case of not sufficiently clarified responsibilities and missing space for clarifying communication. Furthermore, there was double occupancy of the room used for BFB treatment, which could not be avoided and resulted in canceling the BFB session. Moreover, it turned out to be a challenge implementing this new treatment in the nursing team so that all BFB practitioners reached the same level of competence and acquire background knowledge. Further, some problems occurred while ordering materials. In addition, eight BFB practitioners named time management as challenging due to frequently changing TAU schedules. There was not enough time scheduled for an appointment to accommodate patient delays as well as deal with technical issues. Therefore, it was not always possible to keep appointments due to a lack of time and increased workload. Structural problems also existed regarding personnel resources. Due to staff shortages, it was not possible to perform all scheduled BFB treatments. There were also missed appointments due to illness or vacation.

*“So, for example, I was alone on the ward for quite a few days, I just could not keep the appointments.”* (Interview 2: 10).

The increased workload due to BFB treatment presented a strain to the nursing team, as the workload could not be appropriately accommodated at the given time.

*“We all are involved in other routine processes, and this had to be mastered in addition, so to say. And, of course, that has also led to resistance.”* (Interview 10: 9).

Managing the staff was described as challenging during implementation, especially countering frustrations and maintaining staff motivation (2 of 10 BFB practitioners).

*“Yes (...). Well, you have to bring everyone together. And it’s always the case that one person is more motivated than the other. (...) That you find a common ground there.”* (Interview 9: 13).

Moreover, one employee mentioned that the Sars-CoV-2 pandemic led to particular everyday challenges due to constantly changing regulations and increased workload, which made implementing a new therapy much more difficult.

Additionally, the relationship with the patients might have been impaired due to the insecurities of BFB practitioners in performing the novel treatment, which they had no prior experience with. However, most BFB practitioners (8 of 10) felt that BFB treatment did not impair the relationship with patients.

#### 3.2.4. Joy

Seven BFB practitioners enjoyed the implementation of BFB treatment because they learned something new, the benefits for the patients were observable, and the calm relaxation environment created a pleasant working. Frustration arose at times when organizational and structural problems occurred.

*“Yes, it was fun. In the beginning, I was a bit skeptical. Something new is always like that... you have to look first. But when you see that the patients do benefit from it (…), then it’s fun, sure.”* (Interview 5: 26).

#### 3.2.5. Suggestions for improvement

As technical suggestions for improvement the change of the software and the creation of more modern feedback (videos, graphics) were mentioned.

*“I think these video sequences could be a little more modern, the graphics could be a little cleaner, smoother. It still looks so much like 80s/90s software somehow. Then I think it would be more appealing (...) for the patients.”* (Interview 8: 18).

Another suggestion for improvement relates to the own competencies. Nine BFB practitioners would like to have more intensive training and an official advanced training. Furthermore, self-experience sessions are desired, in which BFB treatment can be tried by oneself. In this way, more background knowledge could be acquired and thus self-confidence could be increased.

*“I could imagine doing such a training (...). Basically, to see what else is possible in the bio/neurofeedback area and also to improve my theoretical background knowledge again, (…) that there is a better outcome for the patients when they have a treatment with me (...).”* (Interview 8: 20).

Furthermore, two BFB practitioners would like to have a second BFB device so that more patients can train in parallel. This would lead to an increase in efficiency. In addition, three BFB practitioners wish more support, e.g., in the form of up-to-date manuals and instructions as well as easily reachable contact persons who can help with technical problems or theoretical questions. The compliance of BFB practitioners would be particularly promoted if there was a contact person in their own team.

*“Yes, if something should actually not work, that you could contact someone who can then help in that moment. For example, if we have IT (support) here, if we have computer problems or something. That we can reach someone.”* (Interview 5: 18).

In addition, two BFB practitioners suggest a standardization of the meetings to be able to develop more security and routine. Furthermore, the room used for BFB should be designed more appealing and friendly to support a relaxed training. Regarding appointment coordination, eight BFB practitioners stated that time slots for BFB treatment should be fixed. Furthermore, a calendar should be created to which all BFB practitioners have access to create more transparency regarding scheduling. Regarding personnel planning, one employee suggested to clearly clarify who covers an absentee in case of vacation or illness. Furthermore, an actual-target analysis should be conducted to balance staff resources and BFB sessions offered.

To promote patient rapport and adherence, two BFB practitioners suggested that the first BFB session should include enough time for intensive education and answering of questions. Moreover, it would be beneficial to have the same staff member conducting therapy with a particular patient. Furthermore, there should be a permanent contact person for patients who can be reached on short notice.

*“Actually, we should have a short conversation with the patients. That one schedules a few minutes more time in the first session. We have this information leaflet, but they have so many questions that you actually have to give answers before the first session.”* (Interview 1: 24).

## 4. Discussion

The aim of the present study was to evaluate the implementation process of an individual BFB treatment in addition to the TAU in an inpatient psychosomatic unit. Therefore, the acceptance, satisfaction, feasibility, and ability of implementing this new treatment offer were investigated by applying a convergent parallel mixed methods design. The results of the present quantitative patient assessment exceeded the defined cutoff-value of 23.5 (31) indicating satisfaction of the patients with the BFB treatment. Moreover, based on Bangor et al., system usability suggests a moderate to high acceptance of patients with the BFB treatment (33). The self-generated instrument for measuring acceptance also indicates a high satisfaction and acceptance of patients with the BFB treatment. Most of the patients perceived the sessions as pleasant and expressed their wish to continue BFB treatment after discharge of the clinic. The results of the qualitative interviews with BFB practitioners revealed that most of the BFB practitioners find BFB as suitable for the psychosomatic inpatient unit. The introduction of this treatment enabled the BFB practitioners to expand their own competencies and to take over a therapeutic part of the inpatient treatment of psychosomatic patients. Moreover, BFB treatment allowed BFB practitioners for a different way of relating to the patients resulting in treating the psychosomatic disorder from another perspective. However, the results of the present study revealed several challenges emerging during the implementation process ranging from technical to organizational and structural difficulties. Therefore, technical background knowledge was helpful and partly essential in conducting the sessions. Moreover, not sufficiently clarified responsibilities and a lack of routines showed to be demanding. In addition, tight schedules meant that there was no buffer left for patient delays or spontaneously occurring events. Staff shortages led to cancelling of appointments in case of vacation or illness. However, the biggest challenges revealed to be the increased workload due to additional tasks within the framework of this implementation. The results of both qualitative and quantitative measures show that acceptance and satisfaction with the BFB treatment among both patients and BFB practitioners was high. Although many difficulties were encountered during implementation, BFB practitioners also showed a high level of acceptance. To our estimation, the perceived difficulties during the introduction of BFB are comparable to typically occurring challenges in implementation processes. However, it has become clear that BFB in the psychosomatic-psychotherapeutic inpatient setting must be able to meet special demands. On the one hand, fixed structures narrow the space for novelty. In addition, psychosomatic departments usually treat very heterogeneous groups of patients, to whose different needs one must adapt individually. Typically, inpatient treated patients suffer from a more severe symptom burden than in an outpatient setting. This makes it necessary to adapt the BFB to this special patient clientele.

But how can BFB treatment be implemented in existing daily clinical regimes? Before scheduling BFB treatment, personnel resources should be planned in advance and be available accordingly to minimize the increase of the workload and thus avoid resistance and frustration among the team. Although in this study, the BFB was mainly performed by nursing staff, other patient-related occupational groups could also perform and offer the BFB after appropriate training. The personnel requirements depend on the individual structures of the respective clinic as well as on the number of patients to be treated with BFB. In order to offer adequate BFB training, a time of 1.5 h per patient per week should be calculated, which results from two training sessions of 30 min each as well as 15 min each for preparation and follow-up of the session as well as discussion with the patient. However, an increase in staff is often not feasible due to a shortage of skilled staff nurses and tight budgets. Finding a solution to how BFB treatment can be firmly established in the inpatient setting despite the difficulties and how BFB treatment can be provided permanently, would provide many patients a therapy offer which they could not make use of otherwise, since BFB is mainly offered in the outpatient setting and is considered a self-pay service in most cases. A BFB offer in the psychosomatic-psychotherapeutic inpatient setting now allows access to this type of treatment for a broad patient clientele. In order to ensure a high quality of BFB treatment even though different BFB practitioners perform the sessions, the BFB treatment should be manualized. A standardization and manualization of the sessions would thus facilitate workflows among BFB practitioners. Moreover, an intensive preparation of the BFB treatment and an ongoing support of the BFB practitioners should be ensured. For this purpose, detailed manuals and guidelines should be developed as well as regular training and supervisions should be offered. In addition, a contact person should be available on site and be able to quickly help with both content-related and technical problems. The results of this study also show that appointment management should be discussed with all parties involved before the start of BFB treatment. This would prevent scheduling conflicts and make appointments more reliable.

Due to the challenges identified in this study, we consider a manual based realization and standardization of the BFB treatment for psychosomatic inpatients to be necessary. This would facilitate BFB implementation and BFB practitioners’ workflows, create clear routines, and ensure high quality BFB treatment. Furthermore, this would create the basis for a comparable and effective BFB training in a heterogeneous group of patients. However, a manual based and standardized BFB treatment should not be a substitute for individualized BFB but should be designed specifically for this therapy modality.

### 4.1. Study limitations

Although the results of the present study are promising, some limitations must be considered. First, the selection of patients for the BFB treatment did not follow a structured approach. Since resources and time capacities were limited, it was not possible to offer BFB treatment to all inpatient treated patients. We rather included those patients, who explained interest and had suitable time slots in their therapy schedules. Moreover, four patients discontinued the therapy because they did not like this type of therapy, which leads to a bias in the results. In addition, patient data was lost because some patients were spontaneously discharged from the clinic and were therefore no longer able to complete a questionnaire. Another limitation might be the need for exclusion of patient data due to incomplete questionnaires. Furthermore, the present study did not investigate the effectiveness of BFB treatment. In addition, the treatment success compared to the TAU has not been investigated. It is therefore necessary to conduct appropriate trials in the future.

### 4.2. Clinical implications

The organizational and structural challenges encountered in the inpatient context make clear planning of BFB necessary before the implementation. Not only should space be provided and personnel resources available, but also different areas of responsibility among the BFB practitioners should be clarified. An analysis of the actual situation and the target situation carried out in advance might create a good starting position for determining the feasible scope of BFB treatment.

## 5. Conclusion

The present study shows that BFB as an additional treatment in a psychosomatic-psychotherapeutic inpatient unit is an accepted treatment offer both by patients and BFB practitioners. Even though patient satisfaction and BFB practitioners’ motivation were high, the implementation of BFB treatment in an inpatient context requires special actions to be taken. Therefore, personnel resources should be planned and be available in advance. Moreover, conducting BFB treatment should be standardized to guarantee high quality of the treatment and to simplify workflows for the BFB practitioners. Consequently, the introduction of a standardized and manualized BFB treatment should be considered. Nevertheless, more research needs to be done about suitable BFB protocols for this particular patient clientele.

## Data availability statement

The raw data supporting the conclusions of this article will be made available by the authors, without undue reservation.

## Ethics statement

The studies involving human participants were reviewed and approved by Ethics Committee of the University Duisburg-Essen. The patients/participants provided their written informed consent to participate in this study.

## Author contributions

KS undertook project management, designed the study, actively participated in acquisition of data, performed statistical analysis, interpretation of data, and prepared the manuscript. DB-D made substantial contributions to data acquisition, recruiting of patients, and editing of the manuscript. AK supervised BFB treatment and edited the manuscript. PT, CP, AR, and ND actively participated in implementing the BFB treatment in our wards and contributed to editing the manuscript. E-MS and AB made major contributions to the study’s conception and edited the manuscript. MF actively participated in project management and study design, made major contributions to statistical analysis and interpretation of data, and supervised the preparation of the manuscript and edited it. MT made major contributions to the study’s conception and design, actively participated in the interpretation of data, and revised the manuscript critically for important subject-specific content. All authors contributed to the article and approved the final version of the manuscript.

## Funding

This research received no specific grant from any funding agency in the public, commercial, or not-for-profit sectors. This study was supported by the Open Access Fund of the University Duisburg-Essen.

## Conflict of interest

AK is employed by NeuroFit GmbH.

The remaining authors declare that the research was conducted in the absence of any commercial or financial relationships that could be construed as a potential conflict of interest.

## Publisher’s note

All claims expressed in this article are solely those of the authors and do not necessarily represent those of their affiliated organizations, or those of the publisher, the editors and the reviewers. Any product that may be evaluated in this article, or claim that may be made by its manufacturer, is not guaranteed or endorsed by the publisher.

## Abbreviations

BFB: Biofeedback
NFB: Neurofeedback
PTSD: Posttraumatic stress disorder
TAU: Treatment as usual
PL: Project leader
