# Supplementary material for: Implementing biofeedback treatment in a psychosomatic-psychotherapeutic inpatient unit: a mixed methods evaluation of acceptance, satisfaction, and feasibility
Source: Front Psychiatry. 2023 May 24;14:1140880. doi: 10.3389/fpsyt.2023.1140880 (PMC10244572; doi:10.3389/fpsyt.2023.1140880)
Supplement: Supplementary file 1 [file Table_1.docx]

Supplementary Material

Implementing biofeedback treatment in a psychosomatic-psychotherapeutic inpatient unit: a mixed methods evaluation of acceptance, satisfaction, and feasibility

**Kira Schmidt*, Drazena Barac-Dammeyer, Axel Kowalski, Per Teigelack, Corinna Pfeiffer, Anita Robitzsch, Nora Dörrie, Eva-Maria Skoda, Alexander Bäuerle, Madeleine Fink and Martin Teufel**

*** Correspondence:** Kira Schmidt: kira.schmidt@lvr.de

**A1 Example Schedule for Therapies in the Inpatient Setting**

| **Example Therapy Schedule for Psychosomatic Inpatient Treatment** | | | | | |
| --- | --- | --- | --- | --- | --- |
|  | Monday | Tuesday | Wednesday | Thursday | Friday |
| 07:30 | Check-in 7:30 - 8:00  Nursing support point | Check-in 7:30 - 8:00  Nursing support point | Check-in 7:30 - 8:00  Nursing support point | Check-in 7:30 - 8:00  Nursing support point | Check-in 7:30 - 8:00  Nursing support point |
| 07:45 |  |  |  |  |  |
| 08:00 |  |  |  |  |  |
| 08:15 |  | Creative Therapy  08:15 -09:15  Room 3 |  |  |  |
| 08:30 | Sport and movement therapy  08:30 - 10:00  Room 1 |  | Station meeting PM1  08:30 - 08:55 Room 3 | Time for individual session  08:30 - 09:40 | Time for individual session  08:30 - 09:45 |
| 08:45 |  |  |  |  |  |
| 09:00 |  |  |  |  |  |
| 09:15 |  | Time for individual session  09:15 - 11:00 |  |  |  |
| 09:30 |  |  | Relaxation/Mind-fulness Nursing 09:30 - 10:00 Room 1 |  |  |
| 09:45 |  |  |  | Expressive painting  09:45 - 10:55  Room 3 | Sport and movement therapy  09:45 - 10:45  Room 1/Outdoor |
| 10:00 | Time for individual session  10.00 - 12:00 |  | Time for individual session  10.00 - 12:00 |  |  |
| 10:15 |  |  |  |  |  |
| 10:30 |  |  |  |  |  |
| 10:45 |  |  |  |  |  |
| 11:00 |  | Group therapy Groups I and II  11:10 - 12:00  Room 1 - 2 |  | Group therapy Groups I and II  11:10 - 12:00  Room 1 - 2 | Psychoeducation  11:00 -11.50  Room 3 |
| 11:15 |  |  |  |  |  |
| 11:30 |  |  |  |  |  |
| 11:45 |  |  |  |  |  |
| 12:00 | Lunch break  12:00 - 13:00  Cafeteria on Campus | Lunch break  12:00 - 13:00  Cafeteria on Campus | Lunch break  12:00 - 13:00  Cafeteria on Campus | Lunch break  12:00 - 13:00  Cafeteria on Campus | Lunch break  12:00 - 13:00  Cafeteria on Campus |
| 12:15 |  |  |  |  |  |
| 12:30 |  |  |  |  |  |
| 12:45 |  |  |  |  |  |
| 13:00 |  | Time for individual session  13:00 - 14:15 | Time for individual session  13:00 - 14:00 | Time for individual session  13:00 - 14:00 | Weekly closing / Weekend planning  13:00 -13:30 |
| 13:15 |  |  |  |  |  |
| 13:30 | Room 1 - 2  Concentrative movement therapy  13:30 - 15:00 |  |  |  | Time for individual session  13:30 - 15:00  Time otherwise for:  Exercises on your own |
| 13:45 |  |  |  |  |  |
| 14:00 |  |  | Creative Therapy  14:00 -15:30  Room 3 | Activity Group  13:50 - 15:00  Room 1 |  |
| 14:15 |  | Rounds  Room 3  14:15 - 15:30 |  |  |  |
| 14:30 |  |  |  |  |  |
| 14:45 |  |  |  |  |  |
| 15:00 | Nursing Talks  Time for individual session  15:00 - 16:00 |  |  | Time for individual session  15:00 - 16:00 | Check-out 15:00 - 15:15 |
| 15:15 |  |  |  |  |  |
| 15:30 |  |  |  |  |  |
| 15:45 |  |  |  |  |  |
| 16:00 | Check-out 16:00 - 16:15 | Check-out 16:00 - 16:15 | Check-out 16:00 - 16:15 | Check-out 16:00 - 16:15 |  |

**A2 Patient Satisfaction Questionnaire (according to ZUF-8) – Original German Language**

1. Wie würden Sie die Qualität der Behandlung (Biofeedback), welche Sie erhalten haben, beurteilen?
2. Haben Sie die Art von Behandlung erhalten, die Sie wollten?
3. In welchem Maße hat das Angebot des Biofeedbacks Ihren Bedürfnissen entsprochen?
4. Würden Sie einem Freund / einer Freundin die Biofeedback-Behandlung empfehlen, wenn er / sie ähnliche Hilfe benötigen würde?
5. Wie zufrieden sind Sie mit dem Ausmaß der Hilfe, welche Sie im Rahmen des Biofeedbacks erhalten haben?
6. Hat die Biofeedback-Behandlung, Ihnen dabei geholfen, angemessener mit Ihren Problemen umzugehen?
7. Wie zufrieden sind Sie mit der Biofeedback-Behandlung im Großen und Ganzen?
8. Würden Sie wieder ein Biofeedback-Angebot wahrnehmen, wenn Sie Hilfe bräuchten?

**A3 Patient Satisfaction Questionnaire (according to ZUF-8) – Translated English Language**

1. How would you rate the quality of the treatment (biofeedback) you received?

2. Did you receive the kind of treatment you wanted?

3. To what extent did the biofeedback offered meet your needs?

4. Would you recommend biofeedback treatment to a friend if he/she needed similar help?

5. How satisfied are you with the amount of help you received from biofeedback?

6. Did the biofeedback treatment help you to deal with your problems more appropriately?

7. How satisfied are you with the biofeedback treatment on the whole?

8. Would you use biofeedback again if you needed help?

**A4 Adaptation of the System Usability Scale (SUS) – Original German Language**

1. Ich denke, dass ich dieses Therapieform regelmäßig nutzen möchte.
2. Ich fand das Biofeedback-Training unnötig komplex.
3. Ich fand, dass das Biofeedback-Training einfach durchzuführen war.
4. Ich denke, ich bräuchte die Unterstützung einer technisch affinen Person, um Biofeedback-Training nutzen zu können.
5. Ich fand, dass die verschiedenen Funktionen in dem Biofeedback-Training gut integriert waren.
6. Ich fand, dass es in diesem digitalen Angebot zu viele Widersprüche gab.
7. Ich könnte mir vorstellen, dass die meisten Menschen sehr schnell lernen würden, das Biofeedback-Training durchzuführen.
8. Ich fand das Biofeedback-Training in der Anwendung sehr umständlich
9. Ich fühlte mich bei beim Biofeedback-Training sehr sicher
10. Ich musste eine Menge Dinge lernen, bevor ich mit dem Biofeedback-Training anfangen konnte.

**A5 Adaptation of the System Usability Scale (SUS) – Translated English Language**

1. I think I would like to use this form of therapy regularly.
2. I found the biofeedback treatment unnecessarily complex.
3. I found that the biofeedback treatment was easy to perform.
4. I think I would need the assistance of a technically inclined person to use biofeedback treatment.
5. I found that the different functions in the biofeedback treatment were well integrated.
6. I found that there were too many contradictions in this digital offering.
7. I could imagine that most people would learn to do the biofeedback treatment very quickly.
8. I found the biofeedback treatment very cumbersome to use.
9. I felt very safe doing the biofeedback treatment.
10. I had to learn a lot of things before I could start biofeedback treatment.

**A6 Self-generated Questionnaire regarding Acceptance and Feasibility for Patients – Original German Language**

1. Ich fand die Bearbeitung der Fragebögen in der Vorbereitungsphase als…
2. Ich empfand die Intervention als…
3. Die Intervention war für mich problemlos durchführbar.
4. Die Anforderungen während der Sitzungen empfand ich als…
5. Ich empfand die Rahmenbedingungen während der Intervention als…
6. Ich empfand die Intervention als hilfreich beim Ablenken.
7. Die Ergebnisse der Sitzung auf Papier mitnehmen zu können empfand ich als hilfreich.
8. Ich würde ein Biofeedback-Angebot gerne weiter zuhause nutzen.
9. Ich würde diese Studie weiterempfehlen.

**A7 Self-generated Questionnaire regarding Acceptance and Feasibility for Patients – Translated English Language**

1. I found the processing of the questionnaires in the preparation phase to be...
2. I found the intervention to be...
3. The intervention was feasible for me without any problems.
4. I found the challenges of the session to be...
5. I found the basic conditions during the intervention to be...
6. I found the intervention to be helpful in distracting from thoughts.
7. I found it helpful to be able to take the documentation of the session with me on paper.
8. I would like to continue a biofeedback offering at home.
9. I would recommend this study.

**A8 Semi-Structured Interview Guidelines for Employees – Original German Language**

1. Ist die Biofeedback-Therapie im stationären Setting aus Ihrer persönlichen Sicht eine Ergänzung zu den herkömmlichen Angeboten, kein Ersatz oder genauso oder eher geeignet?
2. Sind Sie im Rahmen der Einführung des Biofeedbacks im stationären Setting auf technische Schwierigkeiten oder Probleme gestoßen?
3. Sind Sie im Rahmen der Einführung des Biofeedbacks im stationären Setting auf Schwierigkeiten oder Probleme bezüglich eigener Kompetenzen gestoßen?
4. Sind Sie im Rahmen der Einführung des Biofeedbacks im stationären Setting auf strukturelle Schwierigkeiten oder Probleme gestoßen?
5. Sind Sie im Rahmen der Einführung des Biofeedbacks im stationären Setting auf Schwierigkeiten oder Probleme in der Beziehungsgestaltung zu Patient:innen gestoßen?
6. Mussten Sie sich im Rahmen der Implementierung des Biofeedbacks besonderen Herausforderungen stellen? Falls ja, welchen? Ist es Ihnen gelungen, die Herausforderungen zu meistern?
7. Konnten Sie durch die Implementierung des Biofeedback-Trainings Ihren eigenen Kompetenzbereich erweitern? Wenn ja, inwiefern?
8. Was könnte bezüglich des Biofeedback-Angebotes im technischen Bereich verbessert werden?
9. Was könnte bezüglich des Biofeedback-Angebotes in Bezug auf Ihre eigenen Kompetenzen verbessert werden?
10. Was könnte bezüglich des Biofeedback-Angebotes im strukturellen Bereich verbessert werden?
11. Was könnte bezüglich des Biofeedback-Angebotes in Bezug auf die Beziehungsgestaltung zu Patient:innen verbessert werden?
12. Hat es Ihnen Spaß gemacht die Biofeedback Therapie durchzuführen?

**A9 Semi-Structured Interview Guidelines for Employees – Translated English Language**

1. From your personal point of view, is biofeedback treatment in the inpatient setting a supplement to the conventional offers, not a substitute, or equally or rather suitable?
2. Did you encounter any technical difficulties or problems during the introduction of biofeedback in the inpatient setting?
3. Did you encounter difficulties or problems regarding your own competences during the introduction of biofeedback in the inpatient setting?
4. Did you encounter structural difficulties or problems during the introduction of biofeedback in the inpatient setting?
5. Did you encounter difficulties or problems in establishing relationships with patients during the introduction of biofeedback in the inpatient setting?
6. Did you have to face special challenges in the implementation of biofeedback? If yes, which ones? Did you succeed in overcoming the challenges?
7. Were you able to expand your own area of competence through the implementation of biofeedback treatment? If yes, to what extent?
8. What could be improved regarding the biofeedback offer in the technical area?
9. What could be improved regarding the biofeedback offer in terms of your own competencies?
10. What could be improved regarding the biofeedback offer in the structural area?
11. What could be improved regarding the biofeedback offer in relation to the relationship with patients?
12. Did you enjoy conducting biofeedback treatment?

A10 Summary of Analysis with Coded Segments and Paraphrases

| Code | Coded Segments | Paraphrase | Summary |
| --- | --- | --- | --- |
| Positive Aspekte\Technik | Ansonsten ist die Ausstattung (...) schon ganz gut  Interview 2: 18 - 18 (0) | Die technische Ausstattung war gut. | Positiv an der Biofeedback-Therapie ist die bisherige technische Ausstattung. Des Weiteren konnte die Beziehung zu Patient*innen positiv beeinflusst werden sowie eine Wirkung der Therapie beobachtet werden. Unterstützend waren dabei Anleitungen und Manuale, Vorgesetzte und Kolleg*innen sowie der Einsatz studentischer Hilfskräfte. |
|  | Aber prinzipiell war es jetzt gut.  Interview 8: 5 - 5 (0) |  |  |
| Positive Aspekte\Wirkung von BF | Scheint ja tatsächlich auch was zu bewirken.  Interview 2: 3 - 3 (0)  Scheint ja was zu bringen  Interview 2: 3 - 3 (0) | Die Biofeedback-Therapie ist wirkungsvoll. |  |
| Positive Aspekte\Unterstützung | Aber sonst fand ich es eigentlich schon gut, dass ich so eine Checkliste da hatte, an die ich mich halten konnte.  Interview 3: 17 - 17 (0) | Checklisten waren eine Hilfe. |  |
|  | Fluch und Segen war natürlich der Einsatz von wissenschaftlichen Hilfskräften. Was natürlich super war, vor allem zur Entlastung für uns von der Pflege.  Interview 8: 9 - 9 (0) | Der Einsatz von studentischen Hilfskräften war eine Unterstützung. |  |
|  | Was aber gut war, dass die Karte da war. Dass man sich die Schritte nochmal anschauen konnte.  Interview 9: 5 - 5 (0) |  |  |
|  | Ja, es gab mit Sicherheit anfangs Kommunikationsprobleme. Missverständnisse glaube ich und Schieflagen und Dysbalancen würde ich das Mal nennen, die aber dann aufgrund der Situation, dass wir ja eine gemeinsame Teamsitzung auch mit den verantwortlichen Vorgesetzten hatten damals oben mit dem Herrn XY und Frau YZ und so und dann da den Verantwortungsbereich klar gestellt haben und auch das notwendige Wissen, den notwendigen Input, den es Bedarf, um sich sicherer zu fühlen, dass das sichergestellt wurde. Ich glaube da wurde schon gut reguliert und auch dann reagiert.  Interview 10: 9 - 9 (0)  das konnte ich ja alles nicht entscheiden das konnte ich nur ansprechen und zur Verfügung stellen, aber dass die Entscheidungsträger also die nächste Führungsebene dann nochmal mitgenommen war und quasi gefordert war eine klare Stellung zu beziehen, ich glaube das war ganz wichtig. Das war sehr unterstützend.  Interview 10: 13 - 13 (0) | Ein Gespräch mit den Vorgesetzten zur Klärung der Verantwortungsbereiche war unterstützend. |  |
| Positive Aspekte\Beziehung zum Pat. | Ich würde eher sagen, dass die Patienten eigentlich ganz gut mitmachen.  Interview 2: 12 - 12 (0) | Die Biofeedback-Therapie hat die Beziehung zu Patient*innen positiv beeinflusst. |  |
|  | Im Gegenteil, ich würde sogar sagen, dass eine Neugier, diese Methode ist nicht bei allen bekannt und dass man darüber auch nochmal einen anderen Kontakt hatte zu den Patienten.  Interview 8: 11 - 11 (0) |  |  |
| Eignung\Geeignet | Es ist geeignet hierfür (…). Genauso geeignet.  Interview 1: 3 - 3 (0) | Die Biofeedback-Therapie kein Ersatz zu den herkömmlichen Angeboten in unserem (teil-) stationären Setting. Es ist eine Ergänzung und genauso geeignet. | Die Mehrheit der Mitarbeitenden (90%) empfand die Biofeedback-Therapie als geeignet für das Angebot unserer Stationen. Dabei sei sie kein Ersatz für herkömmliche Angebote, sondern eine Ergänzung. |
|  | Also ein Ersatz würd ich sagen nicht. Eher eine Ergänzung.  Interview 3: 3 - 3 (0) |  |  |
|  | Ich finde das genauso geeignet.  Interview 4: 3 - 3 (0) |  |  |
|  | Ja, Ergänzung und genauso geeignet.  Interview 5: 3 - 3 (0) |  |  |
|  | Ich würde sagen eine Ergänzung.  Interview 6: 3 - 3 (0) |  |  |
|  | Eine Ergänzung würde ich sagen.  Interview 7: 3 - 3 (0) |  |  |
|  | Das ist eine gute Ergänzung zu den Therapien, die hier angeboten werden.  Interview 8: 3 - 3 (0) |  |  |
|  | Es ist auf jeden Fall eine Ergänzung zur Psychotherapie. Vielleicht sogar besser als manch anderes, aber auf jeden Fall eine Ergänzung.  Interview 9: 3 - 3 (0) |  |  |
|  | Ich würde sagen es ist auf jeden Fall kein Ersatz und es ist eine Ergänzung.  Interview 10: 3 - 3 (0) |  |  |
| Eignung\Nicht geeignet | Finde das Setting schwierig, aber da kommen wir wahrscheinlich noch zu.  Interview 2: 3 - 3 (0)  Scheint ja was zu bringen, nur das Setting ist für mich etwas schwierig.  Interview 2: 3 - 3 (0)  Womit wir wieder beim Setting wären. Inwieweit der Patient, die Patienten da unbeschwert aufspielen können, wenn wir immer daneben sitzen und so einen Kontrollmechanismus haben.  Interview 2: 8 - 8 (0) | Die Biofeedback-Therapie ist für die Patient*innen in diesem speziellen Setting nicht geeignet. | 10% der Mitarbeitenden hielt die Biofeedback-Therapie als nicht geeignet für die Patient*innengruppe dieses Settings. |
|  | Scheint auch nicht für alle unbedingt das Richtige zu sein.  Interview 3: 3 - 3 (0) |  |  |
| Eignung\Nicht beurteilbar | Da fehlen mir eigentlich die Erfahrungen jetzt damit.  Interview 2: 3 - 3 (0)  Also kann ich jetzt noch nichts zu sagen.  Interview 2: 3 - 3 (0) |  |  |
| Probleme\Technisch | und manchmal kam es ja auch in der Handhabung irgendwie, dass mal Fehlermeldungen oder so waren, Kollegen damit nicht gut zurechtgekommen sind. Da musste man schonmal gucken.  Interview 8: 5 - 5 (0) | Manchmal kamen Fehlermeldungen in der Software, mit denen Kollegen nicht gut zurecht kamen. | Probleme in der Durchführung der Biofeedback-Therapie kamen vor allem aufgrund der Komplexität der Technik zustande. |
|  | Also in der Einführungsphase insofern, dass man doch glaube ich länger brauchte, um sich mit der Technik vertraut zu machen als erst angenommen, und dass auch Ängste da waren bei den Mitarbeitern. Also ich selber habe es ja nicht bedient sozusagen. Also ich bin noch gar nicht richtig daran geführt, aber was ich im Allgemeinen wahrgenommen habe, dass es nicht so easy war das umzusetzen. Also dass man es einmal gezeigt bekommen hatte und dann hat es gefunzt, sondern dass man schon ein paar mehr (...) Anwendungen gesehen haben musste, selber gemacht haben musste bevor man da eine gewisse Routine entwickelt hat und sich sicher fühlte.  Interview 10: 5 - 5 (0) | Es braucht viel Übung, um die Technik des Biofeedbacks zu beherrschen. Teilweise kam es zu Sorgen bei den Mitarbeitenden. |  |
| Probleme\Technisch\Software | Wir hatten tatsächlich ein bisschen ein Problem. Software mäßig.  Interview 2: 18 - 18 (0) | Es gab Software-Probleme. | Es traten Softwareprobleme in der Darstellung sowie nicht nachvollziehbare Fehlermeldungen auf. |
|  | Also das Computerprogramm fand ich etwas umständlich. Also so ähnlich, (...) wie unser KIS, wir sind ja schlechte Software hier gewöhnt. War etwas schwierig damit umzugehen.  Interview 3: 5 - 5 (0) | Es ist nicht einfach, mit der Software umzugehen. |  |
|  | Ja, manchmal im technischen Bereich, wo das dann manchmal darstellerisch nicht gut (ist), wo dann halt irgendetwas auf dem Monitor nicht zu sehen war, wo wir dann irgendwie auch erstmal ein bisschen rumgeklickt haben, bis man dann zu dem kam, was man eigentlich kennt. (…) Das kam aber von 10 Mal einmal vor (...).  Interview 4: 5 - 5 (0) | Es kommen Probleme in der Darstellung der Software auf. |  |
| Probleme\Technisch\Gerät | Einmal gab es (mit dem Anschluss) Schwierigkeiten. Ob es letztlich am Gerät lag oder es falsch angelegt war.. Einmal war es, dass ich es nicht durchführen konnte.  Interview 1: 6 - 6 (0) | Es gab Schwierigkeiten, das Biofeedback-Gerät anzuschließen. | Des Weiteren stellte es sich als schwierig heraus, das Biofeedback-Gerät korrekt anzuschließen. Es kam zu Problemen mit dem Gerät, die jedoch durch erneutes Anschließen oder Aufladen behoben werden konnten. |
|  | Ich hatte persönlich einige Probleme mit dem (...) Biofeedbackgerät. Das ist dann von anderen immer lösbar gewesen durch viel längeres Aufladen oder den Stecker nochmal rein und raus. Das war etwas umständlich irgendwie.  Interview 3: 5 - 5 (0) | Es gab Probleme mit dem Gerät, die durch längeres Aufladen oder erneutes Anschließen lösbar waren. |  |
|  | Ja, schon. Also manchmal funktioniert das Gerät halt nicht unbedingt immer. Aber wenn man das dann nochmal ausschaltet, funktioniert das meist beim Einschalten wieder.  Interview 6: 6 - 6 (0) |  |  |
| Probleme\Technisch\Sonstiges | Es gab tatsächlich am Anfang Schwierigkeiten (…) im technischen Bereich. Speziell eher wie man das ganze abspeichert und ausdruckt.  Interview 1: 6 - 6 (0) | Es gab Probleme beim Speichern der Sitzungen sowie beim Ausdrucken. | Zudem war das Speichern und Ausdrucken schwierig. |
|  | Manchmal mit dem Speichern. Ich weiß jetzt nicht bei wem das jetzt vorgekommen ist beim Speichern, aber manchmal war das so, dass der falsche Name ausgewählt war auf dem Dokument. Also nicht die Beschreibung, die man dann eingibt, sondern dass der falsche Patientenname ausgewählt war.  Interview 6: 6 - 6 (0) |  |  |
| Probleme\Eigene Kompetenzen\Up-to-date bleiben | Und jetzt muss man sich da wieder neu reindenken.  Interview 2: 10 - 10 (0) | Es ist schwierig, sich immer wieder neu in die Biofeedback-Therapie reinzudenken. | Die Mitarbeitenden stießen an die Grenzen ihrer eigenen Kompetenzen, wenn sie die Biofeedback-Therapie aufgrund von Krankheit oder Urlaub längere Zeit nicht durchführten. Änderungen im Ablauf konnten so nur schwer integriert werden. |
|  | Also ich war jetzt in der Zeit der Einführung immer mal wieder für längere Zeiträume weg, also nicht damit beschäftigt. Ich habe gemerkt, dass das schwierig (ist) wieder reinzufinden, wenn ich das eine Zeit lang nicht gemacht habe.  Interview 3: 7 - 7 (0)  Das waren dann immer so kleine Steps, die es mir schwierig gemacht haben wieder reinzufinden, weil es kleinere Änderungen immer mal wieder gab.  Interview 3: 7 - 7 (0) | Es ist schwierig, das Biofeedback durchzuführen, wenn man es eine längere Zeit nicht gemacht hat. |  |
|  | Ja, dass sich sehr viel schnell verändert hat und man dann immer neu angelernt hätte werden müssen  Interview 7: 5 - 5 (0)  Liegt aber auch an der Anstellung. Ich hab ja eine dreiviertel Stelle, und dann ist man nicht da, dann kommt man, dann ist wieder was neu. Dann macht man zwei Wochen kein Biofeedback, dann ist wieder alles neu. Aber das ist ja bei jeder neuen Implementierung denke ich so.  Interview 7: 5 - 5 (0) | Es war schwierig, Anpassungen der Therapie mitzubekommen, wenn man sie länger nicht durchgeführt hat. |  |
| Probleme\Eigene Kompetenzen\Unterforderung | Also letztendlich ist es ja so, ich finde das eher unterfordernd sich da hinzusetzen eine halbe Stunde und nur auf das Knöpfchen zu drücken (…). Finde ich alles (…) für unsere Arbeit ein bisschen unterfordernd.  Interview 2: 8 - 8 (0)  Ich weiß nicht. (…) Also ich finde es sehr unterfordernd auch.  Interview 2: 8 - 9 (0) | Die Durchführung der Biofeedback-Therapie war unterfordernd. |  |
| Probleme\Eigene Kompetenzen\Organisation | Also im Bezug auf das Organisatorische bin ich halt manchmal an Grenzen gestoßen, weil die Zuständigkeiten nicht so eindeutig geklärt waren und die (...) verantwortlichen Führungskräfte für die Personalressourcen ja nicht so wirklich ein klares Statement abgegeben haben. Da hätte ich mir glaub ich mehr Klarheit und Struktur gewünscht im Vorfeld wie sich das organisatorisch tatsächlich abbildet und welche personellen Ressourcen man dafür benötigt und welche man auch hat.  Interview 10: 7 - 7 (0) | Die Organisation im Hintergrund war sehr schwierig, da Verantwortungsbereiche und Zuständigkeiten sowie Personalressourcen nicht eindeutig geklärt waren. | Des Weiteren war die Organisation im Hintergrund sehr schwierig, da Verantwortungsbereiche und Zuständigkeiten sowie Personalressourcen nicht eindeutig geklärt waren. |
| Probleme\Eigene Kompetenzen\PC-Kenntnisse | Ich bin jetzt nicht so PC begabt, aber, ja, es ist jetzt nicht so schwierig, dass man sagen könnte man könnte das jetzt nicht durchführen.  Interview 5: 7 - 7 (0) | Fehlende Computerkenntnisse waren ein Problem. | Grenzen der eigenen Kompetenz wurden auch bezüglich Computerkenntnisse deutlich. |
|  | Computerkompetenzen vielleicht. IT-Kompetenzen würde ich sagen.  Interview 7: 7 - 7 (0) |  |  |
|  | Also ich sag mal von meinem Alter her bin ich ja nicht so vertraut mit Computern, mit dem Umgang mit Computern im weiteren Sinne, weil das natürlich nicht zu meiner Ausbildung gehört hat, aber man kann sich das aneignen. Auch wenn man grundsätzlich nicht viel Computererfahrung hat.  Interview 9: 7 - 7 (0) |  |  |
| Probleme\Strukturell\Verantwortlichkeiten | und dann da den Verantwortungsbereich klar gestellt haben und auch das notwendige Wissen, den notwendigen Input, den es Bedarf, um sich sicherer zu fühlen, dass das sichergestellt wurde. Ich glaube da wurde schon gut reguliert und auch dann reagiert. Und ich glaube es war anfangs in der Kommunikation ein bisschen schwierig so die einzelnen Positionen zu erfassen, zu analysieren, zu reflektieren und so weiter. Ich glaube dafür waren manchmal nicht ausreichend Räume da.  Interview 10: 9 - 9 (0) | Es gab strukturelle Probleme, da Verantwortungsbereiche nicht geklärt waren. Es gab nicht genug Raum für klärende Kommunikation. | Strukturelle Probleme kamen auf, da Verantwortungsbereiche nicht ausreichend geklärt waren und es nicht genug Raum für klärende Kommunikation gab. |
| Probleme\Strukturell\Raumprobleme/Besetzung | Raumprobleme natürlich. Wie der Raum benutzt oder besetzt ist.  Interview 9: 5 - 5 (0)  (*Ist der Raum nicht so schön?*) Ja, mit der Belegung auch, dass Doppelbelegungen waren  Interview 9: 9 - 9 (0) | Es gab Doppelbelegungen des für die Biofeedback-Therapie genutzten Raumes. | Des Weiteren kam es zu Doppelbelegungen des für die Biofeedback-Therapie genutzten Raumes. |
| Probleme\Strukturell\Implementierung im Team | Einfach das im Pflegeteam zu implementieren. Das ist ja bis jetzt auch immer noch nicht so super rund, einfach was uns betrifft. Also dass alle gleichermaßen fortgebildet sind im Umgang mit dem Biofeedbackgerät, im Umgang mit der Software, aber auch was so theoretisches Hintergrundwissen sag ich mal betrifft.  Interview 8: 9 - 9 (0) | Es ist schwierig, diese neue Therapie im Pflegeteam zu implementieren, sodass alle Kolleg*innen das gleiche Kompetenzniveau erreichen. | Es stellte sich als schwierig heraus, diese neue Therapie im Pflegeteam zu implementieren, sodass alle Kolleg*innen das gleiche Kompetenzniveau erreichen. |
| Probleme\Strukturell\Fehlende Routinen | Und (...) mir (ist) aufgefallen, dass in dem Terminkalender schon darinstand, was zu machen ist, also EMG oder so, dass das aber nicht mit dem übereinstand, was die (Patienten) vorher bekommen hatten. Ich habe dann zu den Leuten gesagt „Super, heute machen wir ein EMG“, die wussten gar nicht was das war und meinten die hätten vorher irgendwie was anderes gehabt.  Interview 3: 9 - 9 (0) | Es fand keine eindeutige Kommunikation bezüglich der individuellen Trainingsart der Patient*innen statt, sodass es zu Missverständnissen kam. | Das Fehlen von Routinen führte zu Missverständnissen und nicht klar definierten Abläufen. |
|  | Fluch und Segen war natürlich der Einsatz von wissenschaftlichen Hilfskräften. Was natürlich super war, vor allem zur Entlastung für uns von der Pflege, aber auch zeitgleich, dass da nicht so (eine) Routine entstanden ist einfach.  Interview 8: 9 - 9 (0) | Eine fehlende Routine aufgrund der Unterstützung von Hilfskräften machte die Durchführung schwierig. |  |
| Probleme\Strukturell\Bestellwesen | Naja, im Bestellwesen hat es am Anfang etwas gehakt was die Elektrodenbestellung betrifft  Interview 8: 5 - 5 (0) | Es gab Probleme bei Materialbestellungen. |  |
| Probleme\Strukturell\Zeitmanagement | Am Anfang war es schon sehr schwierig, vor allem bzgl. der Stundenpläne, weil die sich andauernd verändert haben.  Interview 1: 10 - 10 (0) | Die Terminplanung war aufgrund der sich häufig verändernden Stundenpläne schwierig. | Zudem war das Zeitmanagement eine Herausforderung. Die Terminplanung war aufgrund der sich häufig verändernden Stundenpläne herausfordernd. Für einen Termin war zu wenig Zeit eingeplant, um Verspätungen von Patient*innen abzufedern sowie mit eventuellen technischen Problemen umzugehen. Des Weiteren war die Durchführung der Termine aufgrund von Zeitmangel und zu hoher Arbeitsbelastung nicht immer möglich.. Bei Terminkollisionen wurde in der Regel das Biofeedback abgesagt. |
|  | Also ich war z.B. auch ganz viele Tage allein auf der Station, ich konnte halt einfach die Termine nicht wahrnehmen.  Interview 2: 10 - 10 (0) | Die Durchführung der Termine war aufgrund von Zeitmangel und zu hoher Arbeitsbelastung nicht immer möglich. |  |
|  | Es waren Termine in dem Kalender, die Termine waren halbstunden Termine geblockt. Und da hab ich gemerkt, das kriege ich gar nicht hin, das eine halbe Stunde aufzuzeichnen und die Leute anzuschließen, abzustöpseln, zu reinigen und vielleicht noch technische Probleme zu umgehen. Das passte also nicht.  Interview 3: 9 - 9 (0) | Die Terminplanung war zu eng getaktet. Für einen Termin war zu wenig Zeit eingeplant, um die Therapie durchzuführen sowie mit eventuellen technischen Problemen umzugehen. |  |
|  | Und ich habe auch mitgekriegt, dass die Patienten eher ein Einzelgespräch bevorzugen als jetzt ein Biofeedback (-Training). Und wenn jetzt Termine kollidierten haben die halt gesagt „gut, dann sag ich Biofeedback ab und nehme das Einzel natürlich in Anspruch.“ Deswegen, ich fand es halt ein bisschen schade, dass wenn Termine kollidierten, dass dann das Biofeedback hinten rüber gefallen ist.  Interview 4: 9 - 9 (0) | Bei Terminkollisionen wurde in der Regel das Biofeedback abgesagt. |  |
|  | Ja, mit den Terminen ist manchmal schwierig, (...) wenn die Patienten (...) Einzel (-therapie) haben.. dann muss man so ein bisschen hinterher sein. Weil dann verschiebt sich das oder man muss dann umplanen. Dann ist aber schon der nächste Patient dran. Dann könnte man das etwas anders planen oder strukturieren, dass sich die Termine irgendwie nicht überschneiden.  Interview 5: 9 - 9 (0) | Die Planung der Termine war schwierig aufgrund der anderen Therapien der Patient*innen. |  |
|  | Manchmal war es schwierig, die Termine zu koordinieren.  Interview 6: 8 - 8 (0)  Also schon bei der Koordination von den Terminen. Wenn dann da irgendwelche anderen Termine reinfallen. (...) Beim Versuch den Termin dann hinzubekommen, war es schwierig.  Interview 6: 10 - 10 (0) | Die Terminkoordination war schwierig. |  |
|  | Also das war sehr ruppelig teilweise, und Doppeltermine und dann musste ein Termin ausfallen und gerade, wenn ein Patient später kommt, dann verschiebt sich alles, oder wenn ein Einzel darein gefallen ist, also auch da glaube ich muss sich das noch ein bisschen verfestigen (...).  Interview 7: 9 - 9 (0) | Es kam zu Terminkollisionen sowie Zeitproblemen bei Verspätung von Patient*innen. |  |
|  | Bis sowas implementiert ist und die Termine gefunden sind… Das dauert und das war natürlich schwierig.  Interview 9: 9 - 9 (0)  dass Patienten in anderen Therapien verschwunden sind, also doppelt verplant.  Interview 9: 9 - 9 (0) | Es kam zu Terminkollisionen. |  |
| Probleme\Strukturell\Personalressourcen | Also ich war z.B. auch ganz viele Tage allein auf der Station, ich konnte halt einfach die Termine nicht wahrnehmen. Dann hatte ich halt wie gesagt Urlaub, Corona, und dann (..) fehlt mir die Zeit.  Interview 2: 10 - 10 (0)  Ja da kommen wir ja wieder zu der personellen Besetzung und dergleichen. Wir haben ja so ein kleines Team (...) und wollen das ja jetzt auch allen näher bringen... aber man kann halt nicht gut vorausplanen aufgrund der personellen Situation.  Interview 2: 14 - 14 (0) | Aufgrund von Personalmangel war es nicht möglich, alle geplanten Biofeedback-Therapien durchzuführen. | Strukturelle Probleme bestanden auch in Bezug auf die Personalressourcen. Aufgrund von Personalmangel war es nicht möglich, alle geplanten Biofeedback-Therapien durchzuführen. Es kam zudem zu Terminausfällen bei Krankheit oder Urlaub. |
|  | Sobald mal einer ausfällt ist es gelaufen. Und das war auch jetzt der Fall, hatten viele Coronafälle, Krankheitsfälle. Und das ist dann...ja, ich kanns nicht leisten.  Interview 2: 10 - 10 (0) | Aufgrund des wenigen Personals kam es schnell zu Terminausfällen, wenn jemand erkrankte. |  |
| Probleme\Strukturell\Arbeitsbelastung | Da würde ich erstmal sagen, immer wenn was Neues dazu kommt, ist die Arbeitsbelastung grundsätzlich höher. Ob das jetzt bei dem mehr ist als bei anderen, kann ich nicht beantworten. Ist ja immer erstmal mehr, wenn man was einführt  Interview 9: 9 - 9 (0) | Die Arbeitsbelastung war durch die Einführung des Biofeedbacks höher. | Die erhöhte Arbeitsbelastung durch die Biofeedback-Therapie hat zu Widerständen im Team geführt. |
|  | Und das fand ich persönlich ein bisschen schade und das ist aber auch dem geschuldet, dass die Kollegen natürlich (...), also dass wir alle, in anderen Routineprozessen drin sind und das quasi dann noch on top geschultert werden musste. Und das hat natürlich auch zu Widerständen geführt. Also so habe ich das erlebt in meiner Mannschaft, dass wir dann gesagt haben "wie sollen wir das alles schaffen, das können wir doch gar nicht leisten" und ja, ich glaube da war es dann ganz wichtig nochmal mehr strukturell entgegenzusteuern und das aufzugreifen und nochmal zu analysieren und zu gucken wie können die Prozesse besser bedient werden. Weil das hat, glaube ich, auf beiden Seiten für sehr viel Frustration gesorgt.  Interview 10: 9 - 9 (0) | Die erhöhte Arbeitsbelastung durch die Biofeedback-Therapie hat zu Widerständen im Team geführt. |  |
| Probleme\Strukturell\Auswahl der Patient*innen | Und ja die Settings.. Kardiologisch und Essstörung. Welche Pat. nehmen letztendlich daran teil.  Interview 1: 10 - 10 (0) | Es war schwierig zu entscheiden, welche Patient*innen am Biofeedback teilnehmen können. |  |
| Probleme\Beziehung zu Patient*innen\Ja | Ist für mich kein Ersatz (für ein Pflegegespräch). Wie soll ich denn das Bezugspflegegespräch führen, mit einer Zielsetzung oder Aufgabe (...) und ich muss gleichzeitig das Knöpfchen drücken und der Patient ist konzentriert. Wie soll das funktionieren?  Interview 2: 12 - 12 (0) | Die Beziehung zu den Patient*innen wurde negativ beeinflusst, da das Biofeedback kein Ersatz für ein Pflegegespräch ist. | Die Beziehung zu den Patient*innen wurde beeinträchtigt aufgrund eigener Unsicherheiten in der Durchführung der Therapie. |
|  | Ja, weil ich ungerne Unsicherheiten vermittle und ich da viele Unsicherheiten hatte teilweise und es dann so kam, wenn man dann mal einem Kollegen geholfen hat, wenn das mal nicht funktioniert hat, dem Patienten immer wieder Sicherheit vermitteln (musste), obwohl man selber eigentlich unsicher ist, das war dann schon schwierig.  Interview 7: 11 - 11 (0) | Die Beziehung zu Patient*innen wurde aufgrund eigener Unsicherheiten bezüglich der Biofeedback-Therapie beeinträchtigt. |  |
|  | Ja eigentlich würde ich da sagen: kaum. Denn das Wichtigste für den Patienten ist ja abgeholt zu werden. Also erst mal dort wo er ist als unwissender Patient, dass eine ordentliche Aufklärung bekommt. Ich glaube, das ist uns auch allen im Laufe der Zeit ziemlich klar geworden, dass man dafür Kompetenzen braucht. Also wieviel braucht, auch um es dem Patienten dann so zu erklären, dass er da mitgenommen wird, dass er es verstehen kann und ich glaube, dass er auf jeden Fall - so wie ich es von den Patienten gehört habe - schon einen besonderen Profit da mitnimmt, wenn er es dann wirklich intensiv betreibt sozusagen.  Interview 10: 11 - 11 (0) | Eine intensive Aufklärung der Patient*innen über die Biofeedback-Therapie ist essentiell, um die Beziehung nicht zu gefährden. |  |
| Probleme\Beziehung zu Patient*innen\Keine Probleme in Beziehung zu Patient*innen | Nein. Also habe ich nicht erlebt.  Interview 1: 12 - 12 (0) |  | Die meisten Kolleg*innen empfanden die Beziehung zu Patient*innen als nicht beeinträchtig durch die Biofeedback-Therapie |
|  | Eigentlich bei mir nicht. Eher im Gegenteil.  Interview 2: 12 - 12 (0) |  |  |
|  | Nee, nicht negativ, aber auch nicht positiv. Ich habe das eher so als einen relativ kühlen, neutralen, wie ne körperliche Untersuchung empfunden. Man macht da halt einfach so sein Ding. Man spricht da jetzt auch nicht besonders, außer am Anfang vielleicht. Nee (...) eigentlich in keine Richtung.  Interview 3: 11 - 11 (0) |  |  |
|  | Nee, nein.  Interview 4: 11 - 11 (0) |  |  |
|  | Nein  Interview 5: 11 - 11 (0) |  |  |
|  | Nö, eigentlich nicht.  Interview 6: 12 - 12 (0) |  |  |
|  | Nein, gar nicht, Das ist ja (...) unsere tägliche Arbeit hier.  Interview 8: 11 - 11 (0) |  |  |
|  | Also die sind ja letztendlich von dir eingeführt worden, also das Thema wurde vorgestellt und die waren ja gut vorbereitet. Von daher nicht.  Interview 9: 11 - 11 (0) |  |  |
| Besondere Herausforderungen\Sondersituation Corona-Pandemie | Und es war ja auch diese straffe Zeit, wenn du überlegst: 2019/2020. Wir waren in der Corona Krise und ich muss sagen hier war einfach viel los also dieser ganze Trubel. Also man muss diesen Zeitpunkt glaube ich auch nochmal betrachten. Dass es sehr schwierig war und herausfordernd, weil wir als Mitarbeiter egal in welchem Bereich ständig neu einspringen mussten auf die Corona-Strukturen, die sich permanent veränderten. Und das Team war dadurch auch sehr belastet, egal in welchem Bereich, ärztlich pflegerisch, psychologisch. Die Herausforderung war mit Sicherheit auch die Belastung durch diese spezielle Situation der Pandemie.  Interview 10: 15 - 15 (0) | Die Corona-Pandemie führte zu besonderen Herausforderungen bei den Mitarbeitenden, die eine Implementierung einer neuen Therapie deutlich erschwerten. | Die Corona-Pandemie führte zu besonderen Herausforderungen bei den Mitarbeitenden, die eine Implementierung einer neuen Therapie deutlich erschwerten. |
| Besondere Herausforderungen\Mitarbeiterführung | Ja. (…) Naja, man muss ja alle an einen Tisch bekommen. Und es ist immer so, dass der eine da motivierter ist als der andere. (…) Dass man da eine Linie findet.  Interview 9: 13 - 13 (0) | Es war besonders herausfordernd, alle Mitarbeitenden mitzunehmen und als Team zusammen zu arbeiten. | Als besonders herausfordernd während der Implementierung wurde die Mitarbeiterführung beschrieben, vor allem die Motivation der Mitarbeitenden trotz der erhöhten Arbeitsbelastung aufrecht zu erhalten und Frustrationen entgegenzusteuern. |
|  | Ja, in der Mitarbeiterführung. Also die Motivation für mich war die größte Herausforderung und immer wieder die Frustration abzufedern und zu motivieren, dranzubleiben und den Benefit zu erkennen trotz der Mehrbelastung. Also das waren für mich die Herausforderungen jetzt im organisatorischen führungstechnischen Bereich. Weil es schwer war, die Kollegen dann bei der Stange zu halten, sag ich jetzt mal so platt. Ja die Frustration, dass so ein neuer Prozess implementiert wird, was eben nicht so perfekt durchstrukturiert ist, dass man dann immer wieder reden musste oder es Auseinandersetzungen gab. Also und das im Backoffice sag ich so, im Hintergrund, also in meinem Schatten das zu managen war eine besondere Herausforderung muss ich sagen.  Interview 10: 13 - 13 (0)  Und auch dann die Kollegen, also eine Kollegin zu finden, die sagt "Ja, ich hab da total Bock drauf und das interessiert mich (...), ich will mich da wirklich reingeben und ich brauche dies und jenes dafür" (…). Also bis ich den richtigen Mitarbeiter da auch gefunden habe, der sich dann aus dem Team herauskristallisiert hat… das war schon fand ich so ein größerer Geduldsprozess sag ich mal…  Interview 10: 13 - 13 (0) | Eine besondere Herausforderung war, die Motivation der Mitarbeitenden trotz der hohen Arbeitsbelastung aufrecht zu erhalten und der aufkommenden Frustration entgegenzusteuern.  Es war schwierig, einen Mitarbeitenden zu finden, der die Organisation hauptverantwortlich leitet. |  |
| Besondere Herausforderungen\Neues Wissen erlangen | Ist ja sehr viel IT, ist ja viel Technik und da musste ich mich besonders darauf einlassen, dass man da reinkommt.  Interview 7: 13 - 13 (0) | Besonders herausfordernd war es, mit der Technik umzugehen. | Des Weiteren war der Umgang mit der Technik sowie die Einarbeitung in die zugrundeliegende Theorie besonders herausfordernd. |
|  | Ich bin ja Altenpfleger von der Grundausbildung her, sodass so manche anatomische Gepflogenheiten, also so Muskelgruppen und so jetzt nicht Teil meiner Ausbildung gewesen (ist), sodass ich mich da ein bisschen einarbeiten musste, aber das ist kein Hexenwerk gewesen. Konnte ich gut mit umgehen.  Interview 8: 13 - 13 (0) | Die Einarbeitung in die zugrundeliegende Theorie der Biofeedback-Therapie war herausfordernd. |  |
| Besondere Herausforderungen\Arbeitsbelastung | Das war eine Mehrbelastung an Arbeit und das war schwierig das so zu koordinieren.  Interview 3: 13 - 13 (0) | Die Arbeitsbelastung durch die Biofeedback-Therapie war besonders herausfordernd. | Des Weiteren stellt die Arbeitsbelastung aufgrund der Biofeedback-Therapie eine besondere Herausforderung dar. |
|  | Wir fangen jetzt gerade an mit der Pflege mal wirklich einzusteigen und was können wir leisten… das braucht immer seine Zeit.  Interview 9: 13 - 13 (0) | Eine Zielgröße an durchführbaren Therapien zu definieren in Bezug zur Arbeitsbelastung war herausfordernd. |  |
| Besondere Herausforderungen\Zeitmanagement | Außer dem zeitlichen Management…  Interview 2: 14 - 14 (0) |  | Besonders herausfordernd war ebenfalls das Zeitmanagement, vor allem das Schaffen von Zeiträumen und die Koordination der Termine. |
|  | Es hat mich vielleicht herausgefordert Zeiträume dafür zu finden. Also, dadurch dass jetzt die Coronaregeln auch auf der Station etwas gelockert wurden und wieder mehr Angebote (...) eingeführt wurden, dass es schwierig war, neue Zeiträume für diese neue Sache zu finden.  Interview 3: 13 - 13 (0)  Die Patienten haben volle Terminkalender und müssen dann noch irgendwie dafür eingeladen werden. Und (...) von unserer Seite war es dann ähnlich.  Interview 3: 13 - 13 (0) | Es war herausfordernd, Zeiträume für die Durchführung der Biofeedback-Therapie zu finden, da es schon viele Angebote im Stundenplan der Patient*innen gibt. |  |
|  | Also nur so mit der Koordination der Termine.  Interview 6: 14 - 14 (0)  Nur, wenn das nicht so geklappt hat mit den Terminen, aber sonst…. (*Konntest du es lösen?*) Ja, eigentlich schon so im Großen und Ganzen. Manchmal nicht, dann musste der Termin halt mal ausfallen (...).  Interview 6: 14 - 14 (0) | Die Terminkoordination war herausfordernd. |  |
| Erweiterung d. Kompetenzbereichs\Keine Erweiterung | Nein. Weil ich nicht genau weiß, wie es funktioniert.  Interview 7: 15 - 15 (0) | Der eigene Kompetenzbereich konnte bisher nicht erweitert werden. |  |
| Erweiterung d. Kompetenzbereichs\Erweiterung der Kompetenzen | Ist sicherlich interessant, klar. (…) (Ich habe) Hintergründe gelernt, ja das kann man sagen.  Interview 2: 16 - 16 (0) | Der eigene Kompetenzbereich wurde durch Hintergrundwissen über die Biofeedback-Therapie erweitert. | Die meisten Mitarbeitenden konnten ihren Kompetenzbereich erweitern, da eine neue Therapiemethode erlernt wurde. |
|  | Ehm, theoretisch ja, denn ich kann jetzt Biofeedback machen. Ja, ich glaub nüchtern betrachtet habe ich wahrscheinlich meinen Kompetenzbereich erweitert.  Interview 3: 15 - 15 (0) | Der eigene Kompetenzbereich wurde durch die Fähigkeit eine neue Therapie durchführen zu können erweitert. |  |
|  | Ehm, ja. (…) Weil das halt eine andere Herangehensweise ist. Ich bin ja Gesundheits- und Krankenpflegerin und komme aus der Somatik, und das ist dann natürlich eine andere Herangehensweise statt Gespräche zu führen oder irgendwie so. Ist halt einfach die Einstellung der Level und so weiter, dem Patienten das zu erklären und dann letztendlich den Patienten alles machen zu lassen.  Interview 4: 15 - 15 (0) | Der eigene Kompetenzbereich wurde erweitert, da eine alternative Therapiemethode erlernt wurde. |  |
|  | Ja, also die einigen Male, wo ich das machen konnte, ja. Also ist nicht verkehrt was Neues zu machen, was Neues zu erproben.  Interview 5: 15 - 15 (0) |  |  |
|  | Ja, schon. (...) Durch das Kennenlernen der Software (...), wie das Anschließen funktioniert und wie Biofeedback an sich funktioniert. Was das für Effekte hat.  Interview 6: 16 - 16 (0) |  |  |
|  | Ja, auf jeden Fall. Es ist eine neue Therapiemethode für mich gewesen. Ich finde das toll, das ist ja evidenzbasiert.  Interview 8: 15 - 15 (0)  Ich finde auch toll was da passiert, diese Hirnwellen, wofür welche gut sind, was man mit dem Biofeedback trainiert. Auch den Umgang mit so einem technischen Hilfsmittel nochmal dazu. Da konnte ich meinen ganzen Horizont eigentlich erweitern, auch meine Kompetenzen, ja.  Interview 8: 15 - 15 (0) |  |  |
|  | Ja. (*Inwiefern?*) Dass ich diese Methode dazu gelernt habe oder dabei bin zu lernen.  Interview 9: 15 - 15 (0) |  |  |
|  | Also ich würde sagen, ich habe Erfahrungen gesammelt wie Implementierung, also was hakelig ist, was schwierig wird und wo man vielleicht in Zukunft ja besonderes Augenmerk darauf richten muss. Dass man eben alle an einen Tisch kriegt, bevor man so ein Projekt startet. (…) Das habe ich für mich gelernt. Jetzt müssen erstmal alle an einen Tisch, da muss die Bereitschaft abgeklopft werden, es müssen die Kompetenzbereiche klar verteilt werden (…).  Interview 10: 15 - 15 (0) | Die eigenen Kompetenzen wurden erweitert, da nun Erfahrungen gemacht wurden, wie man eine neue Therapie in ein bestehendes System implementiert. Besonders wichtig ist, Kompetenzbereiche klar zu verteilen. |  |
| Verbesserungsvorschläge\Technisch\Biofeedback-Gerät | Also ich weiß nicht, ob man da so einen großen Einfluss drauf hat, dass das mit dem Gerät manchmal nicht so klappt, aber ich weiß nicht, ob man das überhaupt beeinflussen kann oder ob das einfach am Gerät liegt.  Interview 6: 18 - 18 (0) | Ein Verbesserungsvorschlag wäre, dass das Gerät funktioniert. |  |
| Verbesserungsvorschläge\Technisch\Software | Ich glaube nicht, dass man an der Software groß was verändern kann. Das stell ich mir schwierig vor, aber das wäre vielleicht schön.  Interview 3: 17 - 17 (0) | Die Software sollte verändert werden. | Ein technischer Verbesserungsvorschlag sei die Veränderung der Software sowie eine modernere Gestaltung des Feedbacks (Videos, Graphiken). |
|  | Und die Software ist so ein bisschen Oldschool. Ich find diese Video- Sequenzen könnten etwas moderner, die Grafiken könnten etwas sauberer, flüssiger sein. Das sieht noch so sehr nach 80er/90er Jahre Software irgendwie aus. Dann wäre es auch glaube ich ansprechender (...) für die Patient:innen.  Interview 8: 18 - 18 (0) | Das Feedback in der Biofeedback-Therapie in Form von Videos und Grafiken sollte moderner gehalten werden. |  |
| Verbesserungsvorschläge\Eigene Kompetenzen\Mehr Wissen | Tatsächlich hätte ich mir mehr Einarbeitung oder Ausbildung gewünscht.  Interview 1: 8 - 8 (0)  Ja, die Weiterbildung würde ich ganz gerne machen.  Interview 1: 20 - 20 (0) | Es sollte eine intensivere Einarbeitung durchgeführt werden. | Ein weiterer Verbesserungsvorschlag bezieht sich auf die eigenen Kompetenzen. Es sollte eine intensivere Einarbeitung sowie eine offizielle Fortbildung angeboten werden. Des Weiteren seien Selbsterfahrungs-Sitzungen erwünscht, bei denen selbst die Biofeedback-Therapie ausprobiert werden kann. So kann mehr Hintergrundwissen erworben und somit die Selbstsicherheit erhöht werden. Zudem sollen Routinen geschaffen werden, um Abläufe besser beherrschen zu können. |
|  | Ich denke schon. Am Anfang stand ja im Raum, so eine Fortbildung zu haben. Vielleicht auch was, dass man ein Zertifikat in der Hand hat, irgendwas was, womit auch offiziell Kompetenzen erweitert wurden. Das wäre glaube ich eine gute Sache.  Interview 3: 19 - 19 (0) | Es sollte eine offizielle Fortbildung durchgeführt werden, bei der man am Ende ein Zertifikat erhält. |  |
|  | Und ich hätte mir so ein bisschen mehr Einarbeitung (...), Erklärung gewünscht, dass ist dafür da und die Wellen sind dafür da. Ich hab mir das jetzt zukommen lassen per E-Mail und hab mir das dann selbst durchgelesen. Ist auch kein Ding, nur die ersten paar Male wusste ich halt gar nicht, womit ich da zu tun habe. Ein bisschen mehr Anleitung wäre irgendwie ganz gut gewesen.  Interview 4: 20 - 20 (0) |  |  |
|  | Joar, vielleicht, dass ich mich mehr damit beschäftige, also mit dem PC. (…) Ja, vielleicht, dass man etwas mehr Zeit hat, sich damit zu befassen, was man da machen könnte, wenn es mal Schwierigkeiten gibt. Also vielleicht etwas mehr (...) mehr Zeit für die Einarbeitung.  Interview 5: 20 - 20 (0) | Es sollte mehr Einarbeitung stattfinden, um mit dem Computer und der Technik besser umgehen zu können. |  |
|  | Ja, also vielleicht noch mehr Aufklärung über Biofeedback an sich und vielleicht noch ein bisschen mehr über das System. Also, wenn da irgendetwas mit dem Speichern nicht richtig ist, dass man das zum Beispiel ändern kann. Also da einfach noch ein bisschen mehr Wissen. (*mehr Hintergrundwissen?)* Ja.  Interview 6: 20 - 20 (0) | Es sollte mehr Einarbeitung geben, um mit möglichen technischen Problemen umgehen zu können. |  |
|  | Der Wunsch vom Anfang ist immer noch offen: Zu verstehen was da geschieht. Und es auch mal selber austesten zu können, das ist alles noch nicht passiert leider.  Interview 7: 20 - 20 (0)  Denn wenn ich was verstehe, kann ich Sicherheit vermitteln. Wenn ich nur so einen Teilbereich übernehme, wie jetzt „mach das mal“, aber ich weiß nicht warum, mit denjenigen, dann find ich ist es immer schwierig eine Frage zu beantworten oder sowas. Ich muss dann immer sagen „Ja da muss ich noch nachfragen“ und das finde ich immer ganz schwierig.  Interview 7: 20 - 20 (0) | Es sollten eigene Biofeedback-Sitzungen stattfinden, um Selbsterfahrung zu sammeln.  Es sollte mehr Einarbeitung und Erklärung geben, um Selbstsicherer zu werden und diese Sicherheit dem Patient*innen vermitteln zu können. |  |
|  | Ich könnte mir vorstellen, so eine Ausbildung zu machen (...). Einfach um zu gucken, was ist noch möglich im Bio-/Neurofeedbackbereich und auch, um mein theoretisches Hintergrundwissen nochmal zu verbessern, auch damit da nochmal ein besserer Outcome für die Patienten bei raus kommt, wenn die eine Behandlung bei mir (...) haben.  Interview 8: 20 - 20 (0) | Es sollte eine offizielle Fortbildung absolviert werden, um das theoretische Hintergrundwissen zu erweitern und so ein besseres Therapieergebnis für Patient*innen zu erzielen. |  |
|  | (*mehr Schulungen, Fortbildungen, Manuale?*) Ja, alles. Also sobald das langfristig eingesetzt wird, und so, wäre ich auch dafür, dass entsprechende Angebote da kommen.  Interview 9: 20 - 20 (0) |  |  |
|  | Ja, ich würde mir wünschen, dass meine Kollegin da eine ordentliche Fortbildung besucht  Interview 10: 20 - 20 (0) |  |  |
| Verbesserungsvorschläge\Eigene Kompetenzen\Regelmäßige Übung | Ja, wir haben uns für morgen vorgenommen, (...) das in der Gruppe nochmal durchzugehen. Das ist vor allem noch die Durchführung, das nochmal zu manifestieren. Dass man das vielleicht dann aus dem FF dann auch kann.  Interview 2: 20 - 20 (0) | Die Biofeedback-Therapie sollte regelmäßig durchgeführt werden, um Abläufe besser beherrschen zu können. |  |
|  | Ja, ich müsste halt mal dran bleiben. Ich müsste halt mal konsequent sagen "ich mach das jetzt auch mal", damit ich nicht immer wieder so rausfalle aus dem Ganzen.  Interview 7: 20 - 20 (0) |  |  |
| Verbesserungsvorschläge\Strukturell\Zweites Gerät | Vielleicht in Zukunft ein zweites Gerät, wenn es jetzt immer mehr Patienten werden.  Interview 1: 18 - 18 (0) | Es sollte ein zweites Gerät angeschafft werden, damit Patient*innen gleichzeitig trainieren können und so mehr Patient*innen teilnehmen können. | Des Weiteren wünschen sich die Kollegen ein zweites Biofeedback-Gerät, damit mehr Patient*innen parallel trainieren können. |
|  | Und dadurch, dass das jetzt so viel, zeitweise in hoher Frequenz, gemacht wurde, dachte ich, wäre es sinnig, ein zweites Gerät anzuschaffen. Wäre natürlich teuer.  Interview 3: 5 - 5 (0)  Vielleicht könnte ein zweites Gerät angeschafft werden.  Interview 3: 17 - 17 (0) |  |  |
| Verbesserungsvorschläge\Strukturell\Unterstützung | Vielleicht könnte man, es gibt schon Anleitungen und Manuale dafür, vielleicht müsste man da nochmal drüberschauen, ob da Veränderungen nötig sind, vor allem vor dem Hintergrund, dass es da technische Problem mal gab, dass man da irgendwie Eventualitäten mit einschließt. Aber sonst fand ich es eigentlich schon gut, dass ich so eine Checkliste da hatte, an die ich mich halten konnte.  Interview 3: 17 - 17 (0) | Die Anleitungen und Manuale sollten aktuell gehalten werden. Es sollten Lösungen für eventuell aufkommende technische Probleme mit aufgenommen werden. | Zudem sollte mehr Unterstützung angeboten werden, z.B. in Form von aktuellen Manualen und Anleitungen sowie festen Ansprechpartnern, die bei technischen Problemen oder theoretischen Fragen behilflich sein können. Die Compliance der Mitarbeitenden würde besonders gefördert werden, wenn es einen Ansprechpartner im eigenen Team gäbe. |
|  | Ja, wenn mal tatsächlich was nicht funktionieren sollte, dass man jemanden kontaktieren könnte, der dann in dem Moment helfen kann. Wenn wir hier z.B. IT (-Untertützung) haben, wenn wir PC-Probleme haben oder so, dass wir dann jemanden erreichen können.  Interview 5: 18 - 18 (0) | Es sollte ein Ansprechpartner erreichbar sein, der bei IT-Problemen unterstützen kann. |  |
|  | als Multiplikator mir das Wissen vermittelt, was ich brauche und für Fragen dann einfach an der Basis zur Verfügung steht. Also, dass sie quasi zum Fachmann oder zur Fachfrau wird für den Bereich und ich an der Basis quasi einen nahen Ansprechpartner habe, der hier im Team ist, und mir hier immer zur Verfügung steht für solche Sachen. Also das wäre ein großer Profit glaube ich und das würde die Compliance auch fördern glaube ich der anderen Mitarbeiter, wenn einer aus den eigenen Reihen ein Fachmann dafür wird.  Interview 10: 20 - 20 (0) | Es würde die Compliance der Kolleg*innen fördern, wenn es einen Mitarbeitenden im Team gäbe, der intensiv ausgebildet wird und als Ansprechpartner zur Verfügung steht. |  |
| Verbesserungsvorschläge\Strukturell\Standardisierung der Abläufe | Und ansonsten glaube ich würde es schon viel helfen, wenn wir uns dann auf ein relativ festes Konzept eingeschossen haben und das über einen längeren Zeitraum durchführen, damit das dann auch gut geölt funktioniert. Jetzt fand ich es immer wieder etwas sperrig.  Interview 3: 19 - 19 (0) | Es wäre leichter, wenn Abläufe standardisiert und über einen längeren Zeitraum durchgeführt würden. | Zudem sollen die Abläufe der Sitzungen standardisiert werden, um mehr Sicherheit und Routine entwickeln zu können. |
|  | Naja, eine Standardisierung der Sitzung, aber das ist ja in Planung, von daher das wird ja gemacht auch.  Interview 8: 18 - 18 (0) | Die einzelnen Biofeedback-Sitzungen sollten standardisiert werden. |  |
| Verbesserungsvorschläge\Strukturell\Raumgestaltung | Ich finde man könnte einen anderen Raum nehmen, wenn das geht. Ich finde das ist da hinten so eine düstere Ecke, (...) man sieht, dass das so provisorisch ist… (*Was würdest du dir wünschen für nen Raum?*) Auf jeden Fall nicht hinten so in der Ecke, vielleicht kann man den Schreibtisch irgendwie versetzen, dass das irgendwie besser ist, man da vielleicht etwas mehr Tageslicht hat. Ist natürlich schwierig mit dem Monitor, ja, verstehe ich, aber das ist so trist.  Interview 4: 22 - 22 (0) | Der für das Biofeedback genutzte Raum sollte ansprechender gestaltet werden. | Des Weiteren ist ein Anliegen, dass der für das Biofeedback genutzte Raum ansprechender und freundlicher gestaltet wird, um ein entspanntes Training zu unterstützen. |
|  | So vom Raum und vom Klima her finde ich ist es immer verbesserungsfähig. Gerade so eine Entspannungsumgebung, dann können wir glaube ich nicht viel daran machen am Raum für den Patienten. Aber ich glaube da haben wir auch schon viel gemacht. (*Sollen wir den Raum verbessern?*) Das ist ein Sportraum. Wenn ich an Sport denke, entspanne ich mich nicht so direkt und ich sehe halt da immer Sportgeräte. Also mich selber persönlich würde es wahrscheinlich ablenken.  Interview 7: 24 - 24 (0) |  |  |
|  | Ich könnte mir vorstellen, dass der Raum generell noch ein bisschen freundlicher gestaltet werden könnte.  Interview 8: 18 - 18 (0) |  |  |
| Verbesserungsvorschläge\Strukturell\Terminkoordination | Nee, finde ich im Moment ganz gut gelöst. Wir haben jetzt extra im Stundenplan ein Zeitfenster eingebaut für das Biofeedback und ich würd sagen das klappt ganz gut.  Interview 1: 22 - 22 (0) | Ein extra Zeitfenster im Stundenplan für die Biofeedback-Therapie würde die Terminkoordination vereinfachen. | Bezüglich der Terminkoordination sollte es feste Zeitslots für die Biofeedback-Therapie geben. Des Weiteren sollte ein Kalender entstehen, auf den alle Mitarbeitenden Zugriff haben, um mehr Transparenz bezüglich der Terminplanung zu schaffen. So könnten Terminkollisionen verhindert und Termine verlässlicher durchgeführt werden. Zudem sollte sich Terminplanung der Biofeedback-Therapie sollte an den Personalressourcen orientieren, um die Arbeitsbelastung nicht zu erhöhen und so Widerstände und Frustration zu vermeiden. |
|  | zum Beispiel feste Termine für Patienten. Also feste Timeslots, um das nicht immer wieder individuell vereinbaren zu müssen.  Interview 3: 21 - 21 (0) | Es wäre leichter, wenn es feste Termine für jeden Patient*in geben würde. |  |
|  | Verbesserung wäre jetzt halt jetzt nicht unbedingt technisch, sondern mit der Terminkoordination  Interview 4: 18 - 18 (0)  Ein Kalender, wo alle irgendwie reingucken (können) oder wo halt Uhrzeiten geblockt werden für bestimmte Patienten auch für die Therapeuten und so weiter. Dass da nicht einfach irgendetwas reingelegt wird und dann auch auf die Theken gelegt wird und dann stehen da zwei Termine und dann überschneidet sich das um 15min oder so. Ist halt für den Patienten totaler Stress, und halt wie gesagt, fällt das Biofeedback meistens hinten rüber. Irgendwie schade.  Interview 4: 22 - 22 (0) | Es sollte einen Kalender geben, auf den jeder Mitarbeitende Zugriff hat, um Terminkollisionen zu vermeiden. |  |
|  | Also mit den Terminen könnte man vielleicht, also falls sich da was überschneidet, dass man sich da mal im Team zusammensetzt und dann mal schaut, was könnte man für eine bessere Lösung finden, dass die Patienten das auch regelmäßig haben und dass man da nicht immer so schauen muss.  Interview 5: 22 - 22 (0)  Prioritäten setzen. Was machen wir, was könnten wir vielleicht doch verlegen. (*Klären welche Therapie hat Vorrang?)* Ja genau, weil sonst kommen dann Patienten an und sagen „Ja, hier ist ja noch ein Einzel“. Und eigentlich ist (es) ja auch so, dass Einzel (-gespräche) die Priorität (haben). Das müsste man halt wirklich nochmal besprechen und auch abklären. Dann würde es vielleicht auch nicht dazu kommen, dass die Termine sich so überschneiden.  Interview 5: 22 - 22 (0) | Es sollte geklärt werden, welches Therapieangebot im Falle einer Terminkollision priorisiert wird. |  |
|  | Ja, (...) also es ist halt glaube ich schwierig umzusetzen, also wenn es so feste Zeitpunkte wirklich gäbe für jeden einzelnen Patienten, aber das kann ja auch mal sein, dass der dann irgendwie krank ist oder so, aber das könnte man auch nicht so einfach umsetzen. Aber, dass man da irgendwie nochmal guckt, wie das mit den Terminen noch verbessert werden kann.  Interview 6: 22 - 22 (0) |  |  |
|  | Ich glaube wir müssen von der Pflege tatsächlich nochmal gucken, wie wir, wenn zum Beispiel eine wissenschaftliche Hilfskraft wegfällt, wie wir das nochmal besser in unseren Stundenplan integrieren können, so dass es keine Überschneidungen gibt mit anderen Therapien und auch mit der Besetzung, die wir haben, dass es auch handelbar ist.  Interview 8: 22 - 22 (0) | Es sollte eine Zielgröße an durchführbaren Biofeedback-Therapien festgelegt werden, sodass es weder zu Überschneidungen mit anderen Therapieangeboten noch zu hoher Arbeitsbelastung kommt. |  |
|  | Und ich habe auch nochmal mit XY gesprochen, wie oft die Patienten dann doch im Einzel gelandet sind und hier und das. Weiterhin diese Zeitfenster im Stundenplan. Mehr Transparenz, für uns, die Patienten. Dass das alles verlässlicher ist, die Termine.  Interview 9: 22 - 22 (0) | Es sollte mehr Transparenz bezüglich der Terminplanung geben, um Terminkollisionen zu verhindern und Termine verlässlicher durchführen zu können. |  |
|  | Die Terminplanung muss sehr an den Personalressourcen orientiert sein, damit die Mitarbeiter nicht in so eine Überforderungssituation kommen oder auch wieder in so eine Frustration und dann Widerstände entwickeln. Das heißt, man muss sich wirklich klar Gedanken machen wieviel Termine mit wieviel Personal kann gemeistert werden und so gemeistert werden, dass die Kollegen sich auch gut dabei fühlen und nicht in so einen Arbeitsverdichtungsfrust kommen.  Interview 10: 22 - 22 (0) | Die Terminplanung der Biofeedback-Therapie sollte sich an den Personalressourcen orientieren, um die Arbeitsbelastung nicht zu erhöhen und so Widerstände und Frustration zu vermeiden. |  |
| Verbesserungsvorschläge\Strukturell\Personalplanung | Wenn das Optimum da ist, dass wir tatsächlich zwei (Mitarbeitende) im Nachmittagsbereich sind und zwei am Morgen, dann stehen die Chancen schon nicht schlecht.  Interview 2: 24 - 24 (0) |  | Bezüglich der Personalplanung sollte die Vertretungssituation im Falle von Urlaub und Krankheit eindeutig geklärt werden. Des Weiteren sollte eine Ist-Soll-Analyse durchgeführt werden, um Personalressourcen und angebotene Biofeedback-Sitzungen auszubalancieren. |
|  | Nochmal gucken welche Berufsgruppen vertreten wen in Urlaub, Krankheit, … Also es ist mir so klar geworden, dass sich nicht alle Berufsgruppen gegenseitig vertreten können, weil die auch unterschiedliche Zeitfenster haben und das dann man dann ganz schnell Druck ausübt, wenn jemand 15 Patienten betreut und dann muss da vertreten werden... Das geht nicht mit dem Stundenplan der Pflege.  Interview 9: 22 - 22 (0) | Die Vertretungssituation im Falle von Urlaub und Krankheit sollte eindeutig geklärt werden. |  |
|  | Da hätte ich mir glaub ich mehr Klarheit und Struktur gewünscht im Vorfeld wie sich das organisatorisch tatsächlich abbildet und welche personellen Ressourcen man dafür benötigt und welche man auch hat. Also so eine Ist- und Soll-Analyse hätte da glaube ich sehr geholfen.  Interview 10: 7 - 7 (0)  Ja also eine Bewusstheit ist ja da schon entstanden und geschaffen worden, dass es mehr personelle Ressourcen benötigt. Das ist ja im Moment in der Mache, das lässt sich aber alles nicht so rasch umsetzen  Interview 10: 22 - 22 (0) | Eine Ist-Soll-Analyse sollte durchgeführt werden, um Personalressourcen und angebotene Biofeedback-Sitzungen auszubalancieren. |  |
| Verbesserungsvorschläge\Beziehung zu Patient*innen\Adhärenz fördern | Tatsächlich könnte man noch ein kurzes Gespräch mit den Patienten führen. Dass man in der 1. Sitzung ein paar Minuten Zeit mehr einplant. Wir haben ja dieses Infoblättchen, aber die haben so viele Fragen, dass man da tatsächlich ja dementsprechend antworten geben muss vor der ersten Sitzung.  Interview 1: 24 - 24 (0) | In der ersten Biofeedback-Sitzung eines*r Patient*in sollte mehr Zeit für aufkommende Fragen eingeplant werden. | Um die Beziehung zu Patient*innen und die Adhärenz zu fördern, sollte die erste Biofeedback-Sitzung genug Zeit für eine intensive Aufklärung und die Beantwortung von Fragen beinhalten. Es wäre förderlich, wenn derselbe Mitarbeitende die Therapie mit einem bestimmten Patient*in durchführen würde. Des Weiteren sollte es einen festen Ansprechpartner*in für Patient*innen geben, der kurzfristig erreichbar ist. |
|  | und dann vielleicht tatsächlich mit der Aufklärung der Patienten.  Interview 4: 18 - 18 (0)  Ich denke, wenn der Patient eine gute Aufklärung hat, dann geht der da auch nicht so spooky dran. Manche sind sehr neugierig und freuen sich teilweise darauf halt einen anderen Zugang zu finden und manche denken so „ah, das hat was mit dem Monitor zu tun, ich werde kontrolliert, ich werde gemessen“. Das ist so leistungsorientiert. Dass man da einfach dem Patienten in einem Vorgespräch irgendwie sagen kann "Nee, das ist überhaupt nicht leistungsorientiert, das ist eine Konditionierung" und so, und das hat nichts mit ich sammle so und so viele Punkte zu tun.  Interview 4: 24 - 24 (0) | Die Patient*innen sollten eine intensive Aufklärung erhalten, um die Motivation und Compliance zu erhöhen. |  |
|  | Die (Patienten) sollten das Gefühl haben, dass es einen Ansprechpartner gibt, dass wenn die Fragen haben, dass man denen das dann gut und verständlich erklären kann. (…) Dass man die nicht vertrösten muss „Dann warten Sie bis XY wieder da ist“. Dass dann alle so ein bisschen involviert sind und alle was dazu sagen können.  Interview 5: 24 - 24 (0) | Es sollte einen festen Ansprechpartner für Patient*innen geben, der in der Nähe und schnell erreichbar ist. |  |
|  | Ich glaube, wenn der Patient merkt, dass wir da ein bisschen selbstbewusster sind und auch mehr Wissen haben und auch sicherer sind im Umgang, gestaltet sich die Beziehung einfach nochmal ein bisschen besser. Weil da die professionelle Ebene einfach nochmal ein bisschen besser hervorgehoben wird (...). Also ich glaube wenn das steht, ist es auch nochmal eine andere Beziehung auf jeden Fall.  Interview 8: 24 - 24 (0) | Die Beziehung zu Patient*innen könnte durch mehr Selbstsicherheit aufgrund von breiterem Hintergrundwissen bezüglich der Therapie verbessert werden. |  |
|  | Ja, also ich glaube da wäre es ganz gut, wenn dann derjenige das Aufklärungsgespräch übernimmt, der dann auch die erste Anwendung mit dem Patienten macht. Das ist ein heres Ziel glaub ich, dass man verfolgt, aber ich glaube so als Ausblick fände ich das für die Strukturen, für die Beziehung zum Patienten und für die Akzeptanz auch als Einstieg ganz gut. Das wäre jetzt halt meine Idee dazu, weil wenn es um Beziehung geht, darum geht.  Interview 10: 24 - 24 (0)  Natürlich wäre es eine wunderbare Sache wenn immer derselbe Mitarbeiter mit dem Patienten das macht, aber das halte ich für ziemlich unrealistisch. Ja das wäre dann ja schon sag ich mal sehr in die Zukunft gedacht.  Interview 10: 24 - 24 (0) | Die Beziehung zu Patient*innen könnte verbessert werden, wenn derselbe Mitarbeitende sowohl die Aufklärung als auch die erste Sitzung mit dem Patienten/der Patientin durchführt.  Ideal wäre es, wenn derselbe Mitarbeitenden alle Biofeedback-Sitzungen eines Patienten/einer Patientin durchführen würde. |  |
| Freude |  |  | Der Mehrheit der Mitarbeitenden hat die Durchführung der Biofeedback-Therapie Freude bereitet, da etwas Neues gelernt wurde, der Nutzen für die Patient*innen beobachtbar ist und die ruhige Entspannungsumgebung ein angenehmes Arbeitsumfeld geschaffen hat. Frustration kam zu den Zeitpunkten auf, wenn organisatorische und strukturelle Probleme entstanden. |
| Freude\Teils teils | Das war für mich eigentlich neutral. Ich verlass mich da auf eure Expertise, dass es gut ist und es was bewirkt. Ich bin da also nicht abgeneigt.  Interview 2: 26 - 26 (0) |  |  |
|  | Manchmal ja und manchmal nein.  Interview 10: 26 - 26 (0)  Es gab Momente, da hat es Spaß gemacht und es gab auch Momente, die waren halt frustran und man das Gefühl hat, es gerät alles ins Stocken und hier steht ein Gerät und das kann eingesetzt werden, aber (...) wir haben gar nicht die Kapazitäten es umzusetzen. Das tat mir dann alles leid.  Interview 10: 26 - 26 (0) | Teilweise hat die Durchführung des Biofeedbacks Freude bereitet und teilweise hat sie Frustration ausgelöst. |  |
|  | Manchmal hat es mir Spaß gemacht, weil ich bin immer sehr dafür, dass Neues ausprobiert wird  Interview 10: 26 - 26 (0) | Teilweise hat es Freude bereitet, da etwas Neues gelernt wurde. |  |
| Freude\Nein | Nein  Interview 3: 25 - 25 (0) | Die Durchführung der Biofeedback-Therapie hat keine Freude bereitet. |  |
|  | Nein.  Interview 7: 26 - 26 (0) |  |  |
|  | Frust hab ich einfach da, wo man nicht weiterkam und wo man gemerkt hat es kommt nicht ins Laufen, es kommt nicht in Gang, es entstehen Auseinandersetzungen, es entstehen Konflikte…  Interview 10: 26 - 26 (0)  und es gab auch Momente, die waren halt frustran und man das Gefühl hat, es gerät alles ins Stocken und hier steht ein Gerät und das kann eingesetzt werden, aber (...) wir haben gar nicht die Kapazitäten es umzusetzen.  Interview 10: 26 - 26 (0) | Die Durchführung hat teilweise keine Freude bereitet, wenn organisatorische und strukturelle Probleme entstanden. |  |
| Freude\Ja | Ja, doch.  Interview 1: 26 - 26 (0) |  |  |
|  | Also ich find das ganz angenehm, mit einem Patienten in einem Raum zu sein, wo nicht gesprochen wird, ich kann dabei irgendwie dokumentieren. Das ist super.  Interview 4: 26 - 26 (0)  Und man weiß, dadurch dass das "Bitte warten"-Schild draußen an ist, dass da nicht gestört wird. Das finde ich total angenehm. Von daher, finde ich das irgendwie ganz gut.  Interview 4: 26 - 26 (0)  Allerdings mag ich das auch, wenn die Patienten da neugierig drauf sind und sagen dann so „Ja, ich hab was gemerkt“ oder „Nee, ich merk überhaupt nichts“. Ist schon interessant, diese Konditionierung halt.  Interview 4: 26 - 26 (0)  Ja, es hat mir Spaß gemacht  Interview 4: 26 - 26 (0) | Die Durchführung der Biofeedback-Therapie hat Freude bereitet aufgrund der ruhigen Entspannungsumgebung und aufgrund der Neugierde der Patient*innen in Bezug auf diese neue Therapieform. |  |
|  | Ja, es hat Spaß gemacht. Am Anfang war ich schon noch so ein bisschen skeptisch. Was Neues ist ja immer so… muss man erstmal schauen. Aber wenn man dann sieht, dass die Patienten doch davon profitieren (...), dann macht es Spaß, klar.  Interview 5: 26 - 26 (0) | Die Durchführung des Biofeedbacks hat Freude bereitet, da der Nutzen für die Patient*innen beobachtbar ist. |  |
|  | Ja.  Interview 6: 26 - 26 (0) |  |  |
|  | Ja.  Interview 8: 26 - 26 (0) |  |  |
|  | Ja. Doch hat es. Also wenn ich es gemacht habe. Ich habe es ja nicht viel gemacht, aber wenn ich es gemacht habe, hat es Spaß gemacht. Und ich stehe auch dahinter und bin motiviert, dass das weiterläuft.  Interview 9: 26 - 26 (0) |  |  |
|  | Manchmal hat es mir Spaß gemacht, weil ich bin immer sehr dafür, dass Neues ausprobiert wird  Interview 10: 26 - 26 (0) | Die Durchführung der Therapie hat Freude bereitet, da etwas Neues ausprobiert wird. |  |
|  |  |  |  |

**A11**

*Type of feedback each patient received during biofeedback training*

| **Patient** | **Feedback** |
| --- | --- |
| 1 | EMG |
| 2 | EMG |
| 3 | NF |
| 4 | EMG |
| 5 | NF |
| 6 | EMG |
| 7 | EMG |
| 8 | NF |
| 9 | NF |
| 10 | NF |
| 11 | NF |
| 12 | EMG |
| 13 | EMG |
| 14 | HRV |
| 15 | HRV |
| 16 | EMG |
| 17 | EMG |
| 18 | NF |
| 19 | EMG |
| 20 | EMG |
| 21 | HVR |
| 22 | HRV |
| 23 | NF |
| 24 | EMG |
| 25 | NF |
| 26 | EMG |
| 27 | EMG |
| 28 | NF |
| 29 | NF |
| 30 | NF |
| 31 | NF |
| 32 | NF |
| 33 | NF |
| 34 | NF |
| 35 | EMG |
| 36 | EMG |
| 37 | NF |
| 38 | EMG |
| 39 | NF |
| 40 | NF |

*Notes.* EMG *= Electromyography,* NF *= Neurofeedback,* HRV *= heart-rate variability.*
